# Embryonic development of selectively vulnerable neurons in Parkinson’s disease

**DOI:** 10.1038/s41531-017-0022-4

**Published:** 2017-06-26

**Authors:** Miguel A. P. Oliveira, Rudi Balling, Marten P. Smidt, Ronan M. T. Fleming

**Affiliations:** 1 Luxembourg Centre for Systems Biomedicine, University of Luxembourg, 6 Avenue du Swing, Belvaux, L-4362 Luxembourg; 2 Department of Molecular Neuroscience, Center for Neuroscience, Swammerdam Institute for Life Sciences, University of Amsterdam, Sciencepark 904, 1098 XH Amsterdam, The Netherlands

## Abstract

A specific set of brainstem nuclei are susceptible to degeneration in Parkinson’s disease. We hypothesise that neuronal vulnerability reflects shared phenotypic characteristics that confer selective vulnerability to degeneration. Neuronal phenotypic specification is mainly the cumulative result of a transcriptional regulatory program that is active during the development. By manual curation of the developmental biology literature, we comprehensively reconstructed an anatomically resolved cellular developmental lineage for the adult neurons in five brainstem regions that are selectively vulnerable to degeneration in prodromal or early Parkinson’s disease. We synthesised the literature on transcription factors that are required to be active, or required to be inactive, in the development of each of these five brainstem regions, and at least two differentially vulnerable nuclei within each region. Certain transcription factors, e.g., *Ascl1* and *Lmx1b*, seem to be required for specification of many brainstem regions that are susceptible to degeneration in early Parkinson’s disease. Some transcription factors can even distinguish between differentially vulnerable nuclei within the same brain region, e.g., *Pitx3* is required for specification of the substantia nigra pars compacta, but not the ventral tegmental area. We do not suggest that Parkinson’s disease is a developmental disorder. In contrast, we consider identification of shared developmental trajectories as part of a broader effort to identify the molecular mechanisms that underlie the phenotypic features that are shared by selectively vulnerable neurons. Systematic in vivo assessment of fate determining transcription factors should be completed for all neuronal populations vulnerable to degeneration in early Parkinson’s disease.

## Introduction

### Parkinson’s disease (PD), symptoms and pathology

**Fig. 1:**
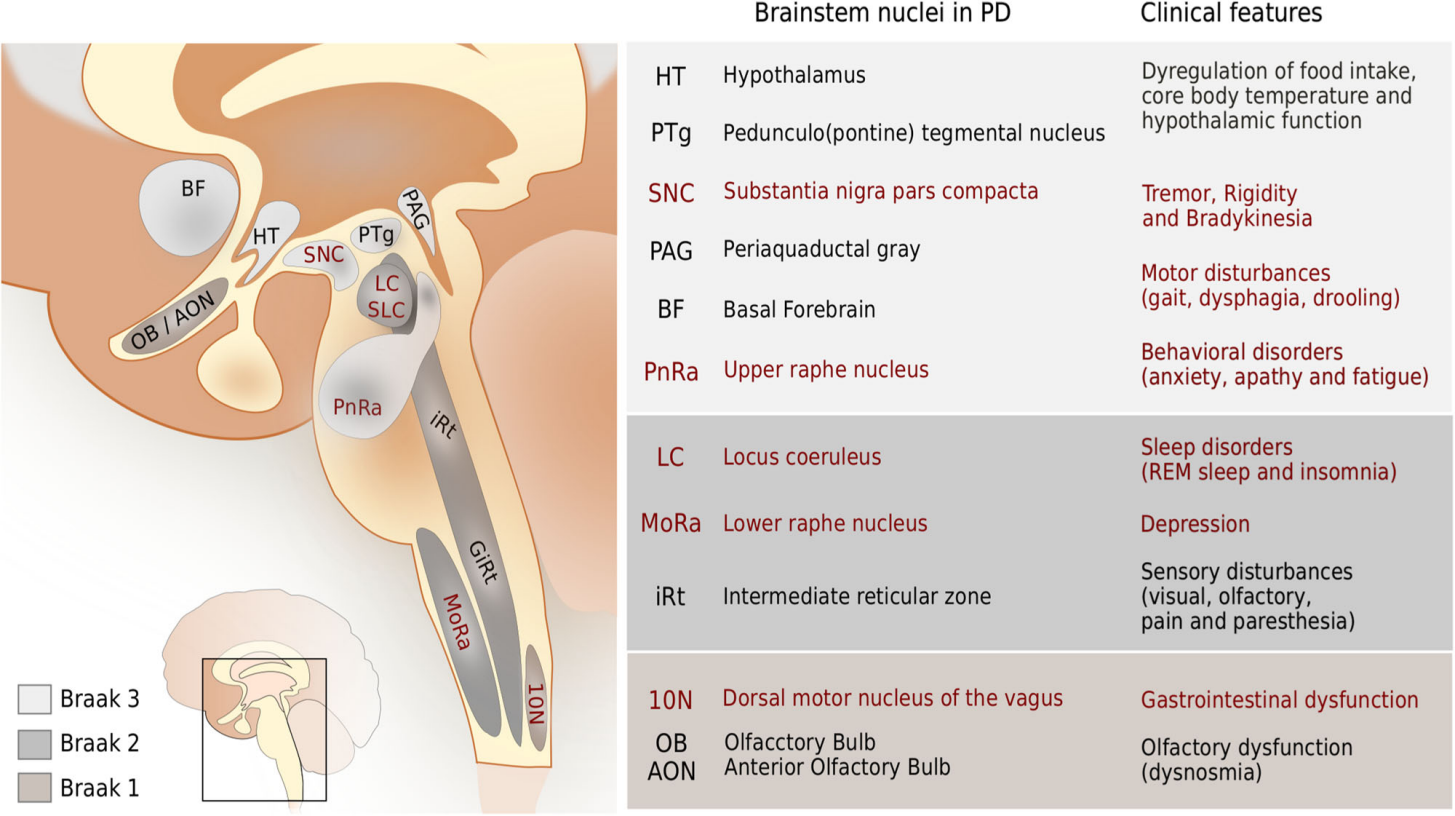
Clinical manifestations,^225–228^ brainstem nuclei, Lewy pathology and cell loss in Parkinson’s disease (PD). Nuclei with evidence cell loss in late PD (*red*) are distinguished from those with evidence for Lewy pathology alone (*black*). The dorsal motor nucleus of the vagus (10N)^7, 8, 18, 229^ and the intermediate reticular zone (IRt) of the medullary reticular formation^18, 230^ are among the earliest brainstem populations with Lewy pathology in PD (Braak stage 1). Located outside the brainstem, both olfactory bulb (OB) and the anterior olfactory nucleus (AON) may also show Lewy pathology at this early stage. Subsequently in Braak stage 2, Lewy pathology is found within three main nuclei: the locus coeruleus (LC),^9, 10^ the lower raphe nuclei (MoRa)^18, 229^ and the gigantically reticular nuclei (GiRt) of the medullary reticular formation.^18^ In Braak stage 3, together with the characteristic motor symptoms (extrapyramidal changes) and degeneration of the SNC,^18, 27, 229^ the upper raphe nuclei (PnRa), located in the pons, also presents with Lewy pathology. In Stage 3, multiple other nuclei of the midbrain tegmentum show Lewy pathology, including the pedunculopontine tegmental nucleus (PTg),^18^ the paranigral nucleus (PaN),^18^ the pigmented parabrachial nucleus (PBP)^18^ and the Edinger Wesphal nucleus (EW).^18^ However, only neuronal cell loss of the SNC is widely considered specific for PD (Supplementary 1). (Figure adapted from ref. 231 with brain ontology according to the Human brain reference atlas of the Allen Brain Atlas.^142^)

PD is a clinical syndrome, identified by a combination of bradykinesia plus resting tremor or rigidity,^1^ that is histopathologically confirmed by identification of both degeneration and loss of dopaminergic neurons (DN) within the substantia nigra pars compacta (SNC).^2^ Neuronal degeneration is characterised by Lewy pathology, which consists of intracellular protein aggregates that co-identify with alpha-synuclein.^3^ The existence of a prodromal phase to PD is supported epidemiologically^4^ by clinical observation of early non-motor symptoms^5^ and by evidence of extranigral Lewy pathology associated with prodromal PD symptoms. The onset of PD is hypothesised to be up to 20 years before the occurrence of motor symptoms, with consistent and early cell loss in the substantia nigra.^6^ In the later stages of PD, cell loss and Lewy pathology is present in other brainstem nuclei^7–10^ but evidence of cell loss in prodromal PD has not yet been reported.^7, 11–13^ Based on the distribution of Lewy pathology in the brain, cardial and cutaneous autonomic nerves,^6^ a neuropathological temporal staging scheme has been proposed for PD.^14–17^ Six sequential Braak stages of Lewy pathology generally seem to coincide with the onset or exacerbation of certain clinical symptoms^18^ (Fig. 1).

Lewy pathology may reflect a compensatory response to proteostatic stress,^12, 19–25^ but may also cause neuronal dysfunction,^26^ e.g., by disruption of axonal organelle transport.^13^ Lewy pathology is also present in other synucleinopathies, e.g., dementia with Lewy Bodies and incidental Lewy Body disease.^13^ Despite variation in the association between Lewy pathology and onset of clinical signs, in the majority of PD patients, non-motor symptoms appear before motor symptoms in a manner consistent with Braak’s neuropathological staging scheme. Prodromal (Braak stages 1, 2) and early PD (Braak stage 3) is characterised by Lewy pathology in a selective subset of brainstem nuclei (Fig. 1). This is consistent with the conclusions of multiple independent studies that have reported cell loss in many of the same brainstem nuclei, albeit in late PD.^7–10, 27^ An anatomically specific and consistent picture of cell loss combined with Lewy pathology provides evidence that certain neuronal populations are selectively vulnerable to degeneration in PD^7, 11, 13^ (Fig. 1).

Selectively vulnerable neurons share some phenotypic characteristics, e.g., unmyelinated axons that have previously been hypothesised to increase the risk of degeneration in PD.^13^ A combination of anatomical, morphological, physiological and biochemical characteristics can be used to define the identity of a neuronal population. Even within a single brainstem nucleus, only certain neuronal populations, identifiable by detailed phenotypic characterisation, may be selectively vulnerable to degeneration. Therefore, comprehensive multimodal phenotypic characterisation of selectively vulnerable neurons in PD is required to further elucidate the relationship between selective vulnerability and shared neuronal phenotype.^28^

**Fig. 2:**
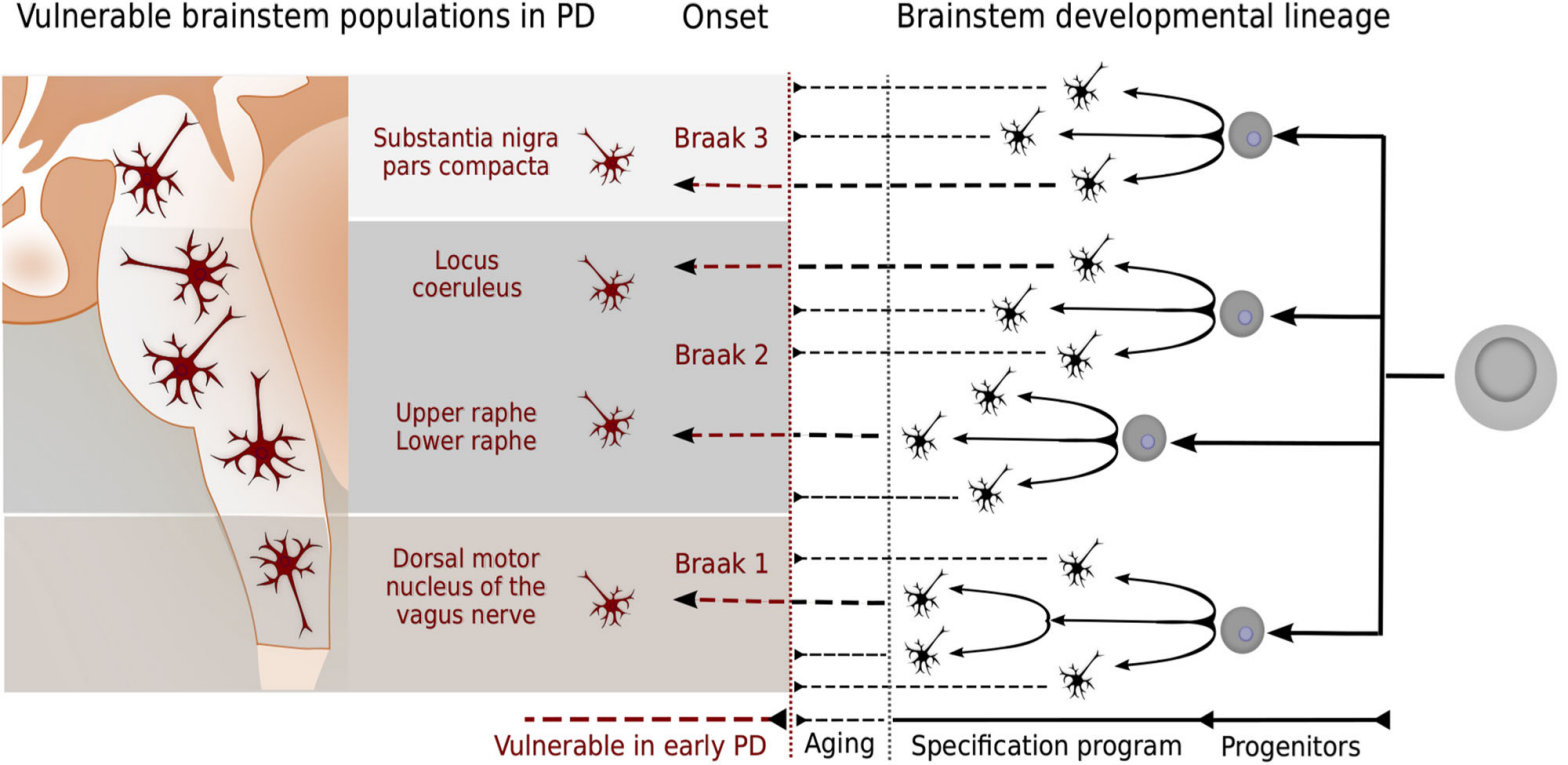
Hypothesis: neurons selectively vulnerable to degeneration in PD share similarities in their cellular and molecular developmental programs. Vulnerable neuronal populations share certain identifiable phenotypic characteristics. Mature neuronal identity is largely the result of a developmental program, that is specific to each cell type. By comparing and contrasting the cellular developmental lineage and requirement for absence or presence of specific developmental transcription factors, we can discover shared developmental similarities of neurons selectively vulnerable in early PD

From an embryological perspective, mature cellular phenotype is the cumulative result of a molecularly specified program that operates on a spatiotemporally evolving developmental lineage. Phenotypically similar neuronal populations share certain aspects of their developmental molecular specification program, spatiotemporal proximity, or both. Therefore, in this review we synthesise the literature on the spatiotemporal developmental lineage position and transcription factor specification of a set of neuronal populations with clear evidence of selective vulnerability to degeneration in prodromal or early PD (Fig. 2). Our objective is to assess whether selectively vulnerable neurons share similar developmental molecular specification programs, spatiotemporal proximity or both.

We chose to restrict our focus to nuclei associated with prodromal evidence of Lewy pathology, evidence of neuronal cell loss in later stages of PD and sufficient developmental literature (e.g., genetic fate mapping). Specifically, we focus on neuronal populations of the dorsal motor nucleus of the vagus (10N), locus coeruleus (LC), upper raphe nuclei (PnRa), lower raphe nuclei (MoRa) and SNC. We summarise (and detail in Supplementary 1) the known phenotypic characteristics specifying the identity of each of the aforementioned mature neuronal populations. For each mature population, we review salient aspects of its developmental lineage and summarise the main transcription factors required for general specification of the corresponding mitotic progenitor, postmitotic progenitor and mature neuron. Where literature permits, we also distinguish between neuronal subtypes within each of these populations based on the origin of the corresponding progenitors and on variations to general specification programs, especially when subtypes are associated with differential vulnerability. We conclude with a discussion of the developmental features that are shared between precursors of vulnerable neuronal populations. This developmental perspective compliments previous efforts to understand the phenotypic characteristics that are shared between selectively vulnerable mature neuronal populations^13^ (Fig. 2).

### Neuronal identity of vulnerable populations

Neuronal identity and its cellular and molecular phenotypic specification is mostly encoded by a profile of transcription factors, expressed by ancestral progenitors and by postmitotic neurons.^29, 30^ These transcription factors are expressed early in the developing brain, downstream of specific developmental inductions, and are responsible for the gradual fate restriction of the embryonic pool of pluripotent stem cells. The enormous variety of neuronal populations arises from combinatorial induction that is specific to each particular location within the brain, where subtle inductive differences generate different neuronal populations.^29^ Lineage tracing studies provide a powerful means to understand the properties of mature populations, their development, homoeostasis and disease vulnerability, especially when combined with experimental manipulation of signals regulating cell-fate decisions.^31^

#### Phenotypic characteristics are shared between vulnerable populations

**Fig. 3:**
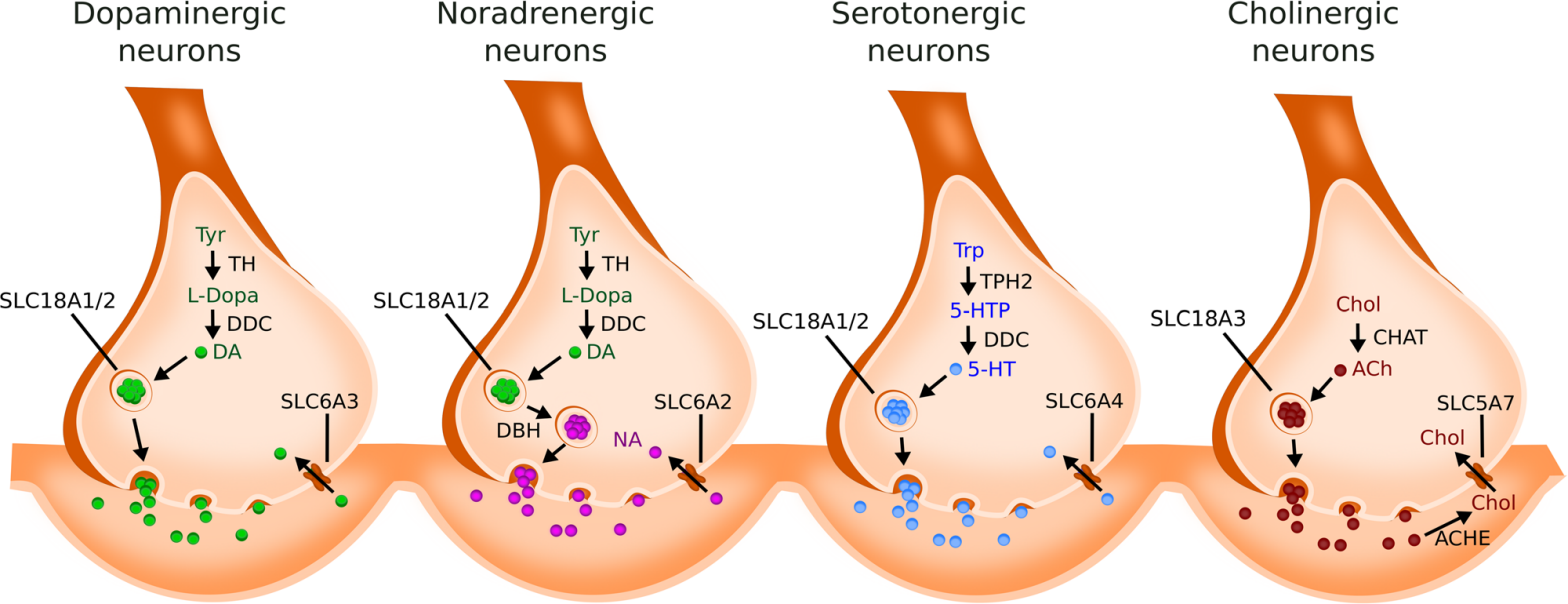
Neurotransmitter identity (adapted from ref. 232). Multiple transmitters and transmitter-like substances have been studied in PD and it is clear that neurons releasing neurotransmitters other than dopamine (DA) are also susceptible to neurodegeneration.^227^ These transmitters include the monoamines serotonin (5-HT) and noradrenaline (NA) and the neurotransmitters acetylcholine (ACh). In catecholaminergic neurons (dopaminergic, noradrenergic and adrenergic), the neurotransmitter is synthesised from the amino acid tyrosine (Tyr) in a common biosynthetic pathway.^232^ Mesodiencephalic DN of the SNC, together with the noradrenergic neurons of the LC, require the expression of tyrosine hydroxylase (TH) and amino acid decarboxylase (DDC). As opposed to DN, noradrenergic populations further require the expression of dopamine beta-hydroxylase (DBH), which converts DA into noradrenaline. Adrenergic neurons, in turn, also require the expression of phenylethanolamine-N-methyl-transferase (PNMT), which converts noradrenaline into adrenaline. On the other hand, although serotonergic neurons from the raphe nucleus synthesise 5-HT through the hydroxylation of tryptophan, a reaction catalysed by tryptophan hydroxylase (TPH), they also require the expression of DDC.^232^ In neurotransmitter packing, both catecholaminergic and serotonergic neurons require the same vesicular monoamine transporters, SLC18A1/2.^232^ The transporter responsible for the re-uptake of neurotransmitters from the synaptic cleft is specific for each population. Serotonergic neurons require the transporter SLC6A4, DN require SLC6A3 and noradrenergic neurons require SLC6A2. For neurotransmitter degradation, the catecholaminergic neurons require catechol-O-methyl transferase (COMT), monoamine oxidase (MAO) and aldehyde dehydrogenase (ALDH), while the serotonergic neurons only require MAO and ALDH.^232^ Cholinergic neurons secrete acetylcholine (ACh), which is synthesised by the choline acetyltransferase (CHAT) from acetyl-CoA and choline (Chol). In cholinergic neurons from the 10N, ACh is packed into synaptic vesicles by an energy-dependent process that involves the SLC18A3. This vesicular ACh is released in the synaptic cleft and is rapidly converted into Chol, by the acetylcholinesterase (ACHE), which then is transported intracellularly by SLC5A7.^227, 233^ (also see Supplementary 2)

It has been hypothesised that selectively vulnerable neurons share a set of common phenotypic characteristics leading to an increased risk of degeneration in PD.^13^ These characteristics, which include neurotransmission, electrophysiology, morphology and connectivity, do seem to be consistent with age being the single largest risk factor in PD.^13^ Regarding neurotransmission, the presence of high levels of cytosolic monoamines is hypothesised to underlie selective degeneration^13, 32^ since these populations also generally include a catecholamine-derived neuromelanin pigment in primates^13, 33^ (Fig. 3 and Supplementary 1 and 2). Electrophysiological characteristics associated with increased risk include autonomous activity, broad action potentials and a low intrinsic calcium buffering capacity.^13^ On morphology and connectivity, vulnerable populations are generally characterised as having long, poorly myelinated, highly branched axons and terminal fields.^13^

Currently, the majority of genes or expression signatures used in the biochemical characterisation of neuronal populations are related to their neurotransmitter identity (Fig. 3 and Supplementary 1 and 2). However, this characterisation only covers the ability to produce, secrete and re-uptake specific neurotransmitters,^34, 35^ which is not sufficient to completely specify the neuronal identity.^34^ Some neurons co-release more than one neurotransmitter^36, 37^ and neuronal plasticity enables neurons to switch between neurotransmitters.^34, 38–40^ Therefore, a more inclusive characterisation of neuronal identity, besides neurotransmission alone, is required^28^ (Supplementary 1).

Developmental programs that specify neurotransmitter phenotypes are well studied aspects of neuronal identity,^28, 29, 41^ particularly with respect to terminal differentiation of monoaminergic neurons (noradrenergic, dopaminergic and serotonergic).^34^ Neuronal transcriptomic analysis is also a powerful way to characterise neuronal identity,^42–44^ e.g., transcriptome sequencing is quantitative, and highly reproducible.^28, 45^ However, the correlation between transcript and protein levels is generally too weak for accurate quantitative inference of one from the other.^46^ Targeted quantification of key developmental proteins and confirmation of their role in specification of multiple nerve cell functions^47, 48^ can be used to compliment genome-scale measurements and lead to a more robust characterisation of neuronal identity.^49^ By inferring cell-type-specific function from developmental programs and expression profiles, one can also assess the cell-type specificity of functional attributes, derived from parallel morphological and electrophysiological studies.^28^

## Results

### Development of vulnerable brainstem populations

In the past two decades, substantial progress in developmental neuroscience has uncovered a large set of extracellular signals and transcriptional regulators that control the development and maturation of different types of neurons. However, the developmental program is not yet fully understood for each and every neuronal population. In order to better understand the generation of different neuronal populations, it is important to study the mechanisms behind the maintenance of infinite self-renewal capacity in stem cells (unrestricted fate potential) and the mechanisms responsible for lineage commitment during differentiation.^50^ The final neuronal phenotype comprises generic pan neuronal characteristics and more specific characteristics, such as origin and termination of axonal projections.^51^

**Fig. 4:**
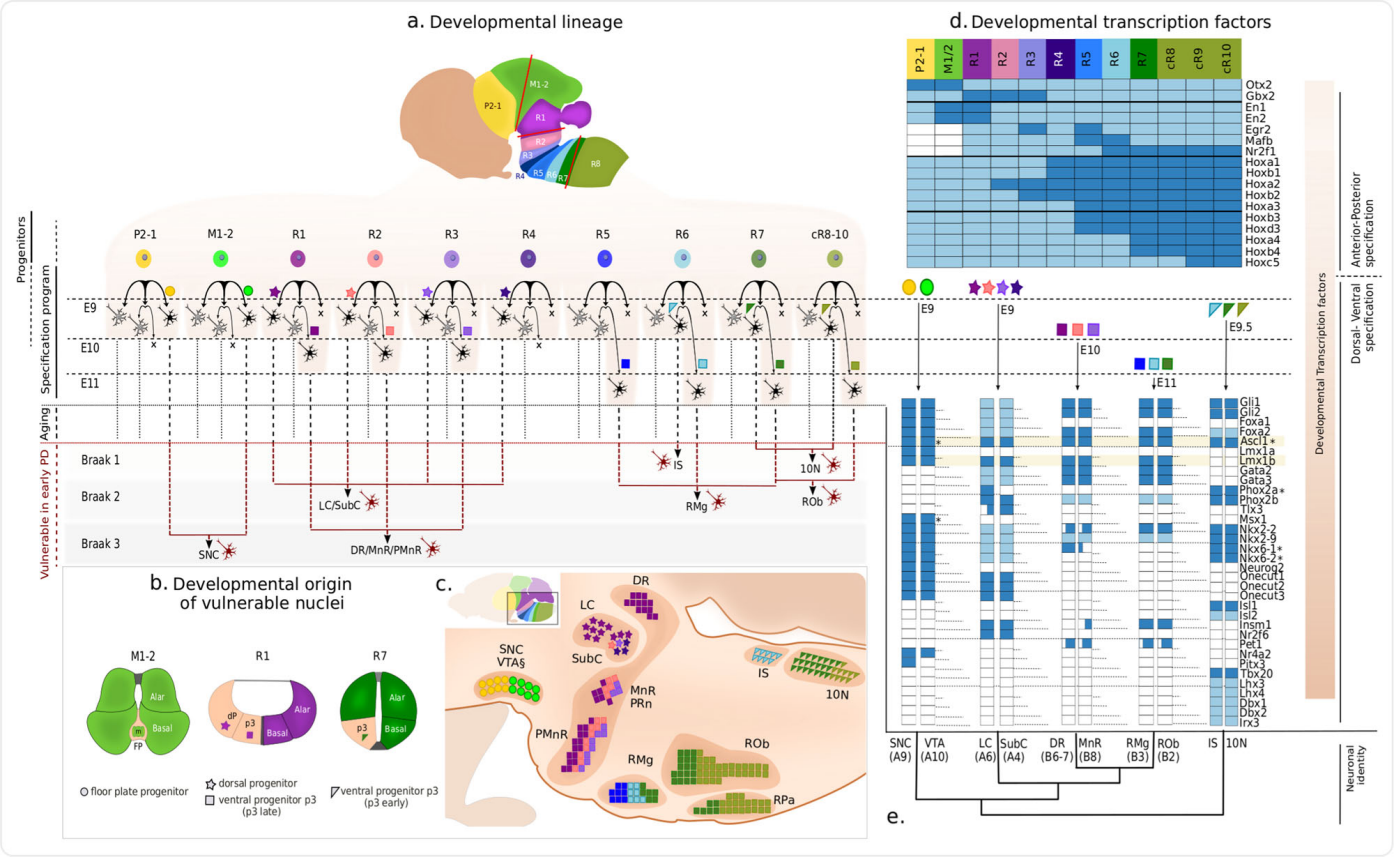
Brainstem development and neuronal specification program of brainstem vulnerable populations in PD. **a** Each brainstem progenitor originates from one rostro-caudally segmented neuromere (colour coded) and gives rise to specific mature neurons via a developmental lineage. **b** Transverse view of three neuromeric segments (positions correspond to *red lines* in **a** with dorso-ventral progenitor origin (shape coded). **c** Mature neuronal populations after development, which may include migration, in vulnerable brainstem nuclei within a quasi-saggital section of a juvenile brain (neuromeric segment is colour coded and dorso-ventral progenitor origin is shape coded, as before), adapted from.^58, 61, 63^ **d** A set of developmental transcription factors is associated with the identity of each segment (*upper*) and the specification program for each neuronal population (*middle*), whether required to be expressed (*dark blue*), or required not to be expressed (*light blue*) or an experimental knowledge gap (*white*). **e** Neuronal populations are clustered by their developmental requirements highlighting similarities. Dorsal motor nucleus of the vagus (10N), medullary/lower raphe nuclei [MoRa (RMg/B3, ROb/B2, RPa/B1)], pontine/upper raphe nuclei [PnRa (PRn/B5, DR/B6-7, MnR/B8, PMnR/B9)], locus coeruleus (LC/A6), subcoeruleus (SubC/A7), substantia nigra pars compacta (SNC/A9). Neuronal populations highlighted in this review are presented in *black* and *red* (vulnerability), the remaining neuronal populations are in *light grey*. The X sign represents the absence of neurogenesis from the corresponding progenitor. *involved but not required. **medial position. See Supplementary Information for details, including references to supporting literature

Neurons originate from multipotent stem cells in the neural plate (Supplementary 3.1) that continuously limit their fate and generate restricted mitotic progenitors that, in a sequential order, give rise to neuronal and glial progenitors.^52–54^ Neuronal differentiation occurs at different embryonic stages (E) and within different neuromeric segments of the early brain (prosomeres P3-1, mesomeres M1-2 and rhombomeres R1-8) (Fig. 4). For each neuromere-specific neuronal progenitor, the induction of a specific neuronal fate is controlled in a context-dependent manner by a combination of intrinsic factors and extrinsic signalling molecules, both of which act as regulators of neuronal differentiation^52–55^ (Supplementary 3.2). Specific intrinsic factors and inductive combinations result in the upregulation (or downregulation) of certain genes, mostly transcription factors, which are required to be active (resp. inactive) to ensure lineage commitment and generation of specific neuronal fates.^50^ Developmental transcription factors can either be transiently or constitutively expressed during development, and still be individually required for fate restriction and the generation of specific mature populations.

Within each neuromere, an initial set of active genes, activated before neurogenesis, confers an intrinsic segmental identity to the corresponding progenitors (Fig. 4), which is inherited or diversified during neuronal induction by extrinsic signals, especially during terminal differentiation^56^ (Fig. 4d). An anatomically defined nucleus may have different neuronal subtypes with differences that can be traced back to the neuromeric origin of the corresponding progenitor. For example, genetic fate mapping of raphe nuclei has established that different serotonergic neuronal subtypes arise from separate rhombomeres and from variations of the general serotonergic specification program.^44, 48, 51, 57–62^ The same developmental principle also applies to other types of neuron, e.g., noradrenergic^63^ and visceromotor neurons,^64, 65^ where nuclear subtype specification has also been associated with neuromere-specific transcription factors and variations of the general specification program for each type of neuron. For mesodiencephalic dopaminergic populations^66–78^ mediolateral progenitor positioning has a similar effect.

#### Visceromotor populations of the dorsal motor nucleus of the vagus

Visceromotor neurons are a subset of cranial motor neurons that project from multiple brainstem nuclei, including the 10N, towards internal organs, like lungs, heart and vicera. The 10N visceromotor population project their axons to the viscera, via the vagus nerve (Supplementary 1.1).

##### Progenitors of visceromotor neurons

Multiple subsets of visceromotor neurons are generated throughout brainstem, except R1.^64, 79^ These subsets originate from neuromere-derived basal p3 progenitors (p3 or pMNv), which bilaterally flank the floor plate.^64, 65, 80^ These progenitors are also common to both branchiomotor^81^ and serotonergic populations.^57, 82^ The p3 pool of progenitors is generated after an anteroposterior (AP)-graded retinoic acid (RA) signalling, which confers multiple rhombomeric identities, specified by a combination of *Hox* genes^65^ and a ventral SHH signalling, which is necessary to impose a specifically motor fate^64, 83^ (Fig. 4d and Supplementary tables 1, 2).

##### General specification program of visceromotor neurons

The general specification program of visceromotor neurons (and branchiomotor neurons) begins with the acquisition of the correct p3 progenitor identity and neurogenesis around E9.5 (Embryonic day 9.5)^57, 82^ (Fig. 4d and Supplementary table 1-2). Visceromotor neurons require simultaneous expression of *Nkx2-2* and *Nkx2-9*.^64, 65, 81, 84^ *Nkx6-1* and *Nkx6-2* are also expressed at this stage but this expression is not necessary for specification, despite their importance in repressing alternative interneuronal fates and addressing migration and pathfinding.^64, 65^

*Phox2b* is expressed earlier than *Phox2a* (not required) and *Phox2b* expression is required for visceromotor specification,^57^ since *Phox2b*-mutant mice are depleted of all visceromotor and branchiomotor neurons.^85^

In order to generate a motor neuron phenotype, p3 progenitors require low or absent *Foxa2* expression.^57^ From E10.5 onwards, and within the rhombomeres R2-3 and R5-8, some p3 basal progenitors switch their visceromotor fate towards serotonergic, which coincides with the up-regulation of *Foxa2* and the down-regulation of *Nkx2-9* and *Phox2b*.^57, 82^

In the visceromotor specification program, *Ascl1* is expressed but not required.^86^ Post-mitotic motor neurons require the expression of *Isl1*,^65, 87, 88^ and *Tbx20* is only expressed within branchiomotor and visceromotor neurons,^65, 89^ like the 10N visceromotor neurons. Both *Lhx3/4* are not expressed in the 10N visceral motor neurons, since they are key determinants of the ventral pathway in motor neurons and this population has a dorsal orientation of their axonal projections.^65, 90^ Developing visceromotor neurons do not express genes characteristic of dorsal progenitor sub-types, such as *Dbx1/2* ^91^ and *Irx3*.^92^ Also, their specification is not dependent on the somatic motor neuron markers *Pax6*,^65, 93^ *Mnx1*,^65, 94^ *Olig2*,^65, 92^ or *Isl2*.^65, 87, 88^

##### Subtype specification program of visceromotor neurons

In early PD, Lewy pathology,^7, 11^ but not cell loss, has been reported in the inferior salivatory nucleus (IS), which is adjacent to the 10N, and projects visceromotor neurons within the glossopharyngeal nerve. In contrast, Lewy pathology is more pronounced and is consistently observed in the 10N.^11^ Visceromotor neurons from the IS and 10N nuclei both originate from basal p3 progenitors and during development they migrate dorsally into an alar position^64, 65^ (Fig. 4a, b). Although these subtypes share the same general specification program, they have different rhombomeric origins. The visceromotor neurons from the IS originate from R6, while those from the 10N originate from R7-8.^64, 65^ IS progenitors require the *Mafb* gene to be present and the *Hoxa4* and *Hoxb4* genes to be absent, whereas 10N progenitors require the opposite (Fig. 4d). To our knowledge, no subtypes have been reported based on variations of the general specification program described above.

#### Serotonergic populations of raphe nuclei

Raphe nuclei contain the central serotonergic populations (B1–9),^95^ totalling 20,000–30,000 neurons in rats,^95^ which are distinguishable by their anatomical location, and can be divided into caudal (B1–B4) and rostral clusters (B5–B9). Raphe nuclei clusters^61, 96^ are positioned caudal and rostral to rhombomere R4, which is a neuromeric segment that is only populated by motor neurons.^82^

##### Progenitors of serotonergic neurons of raphe nuclei

Raphe nuclei contain serotonergic neurons generated from a mixture of rhombomere-derived basal p3 progenitors, which initially reside in the bilateral paramedian territories that flank the floor plate^48, 51, 58, 61, 97^ (Fig. 4a, b). These p3 progenitors are common to brainstem viscero-motor neurons and branchio-motor neurons,^57, 82^ with the exception of those that are R4-derived.^48, 58, 61^

A multipotent pool of hindbrain p3 progenitors are differentially induced along the AP axis, due to a gradient signalling by RA, FGF8 signalling at rostral positions, FGF4 signalling at caudal positions and ventral SHH signalling.^98^ Differential combinatorial induction across the hindbrain determines the neuronal sub-type specification program,^51, 61^ via a rhombomeric-specific expression of *Hox* genes (Fig. 4d and Supplementary tables 1, 2). Differential expression of *Hox* genes, the *Hox code*, results in considerable differences between caudal and rostral serotonergic profiles. Caudal serotonergic populations, which localise within B1–B4 populations, result from progenitors expressing multiple *Hox* genes, while the rostral serotonergic populations within B1–B4 populations result from progenitors highly expressing *Hmx2/3* genes (Fig. 4d upper table). Both Shh signalling at ventral positions^99^ and rhombencephalic absence of *Otx2* expression are required for the correct development of a serotonergic phenotype.^61, 100^

##### General specification program of serotonergic neurons of raphe nuclei

The general specification program for serotonergic neurons is known^96, 101^ although the details of the molecular mechanisms are still poorly understood.^58, 60, 61, 102–105^ In mice, the rostral and caudal cluster of serotonergic neurons start to differentiate at E10 and E11, respectively^51, 57, 82^ and their morphology is only defined after the P0 stage. The serotonergic developmental program mostly occurs through the activation of a pair of genetic cascades^51, 57, 98, 106^ (Fig. 4d and Supplementary tables 1, 2). The first cascade involves ventral SHH signalling and subsequent activation of both *Nkx* transcription factors (*Nkx2-2*) and *Foxa2* expression, which results in activation of *Gata* transcription factors (*Gata2/3*) and *Lmx1b*.^51, 61, 100^ This specification program requires the absence of *Otx2* expression and the presence of *Nkx2-2* expression.^51, 84^ Importantly, there is also a parallel secondary cascade, where *Ascl1* expression is activated around E11.5^82, 107^ and instructs a sequential expression of *Gata3, Lmx1b*, and *Insm1*.^51^ *Ascl1* and *Insm1* are both part of the genetic regulatory network that controls serotonergic identity,^108^ where *Insm1* expression contributes with an additional control of THP2 expression.^51^ On the other hand, *Lmx1b*, which is expressed downstream from Gata2/3 in both raphe clusters, is required for terminal differentiation and maintenance of all serotonergic populations. Like all aminergic neurons, *Lmx1b* has been suggested to regulate the expression of the vesicular monoamine transporter.^51, 109^ *Lmx1b* might also regulate SLC6A4 (SERT) and TPH2 expression^110^ in mature populations.

It has also been described that a complete neurochemical serotonergic phenotype is, to a certain extent, controlled by *Pet1*.^51^ Both *Lmx1b* and *Pet1* have been implicated in the regulation of SLC6A4^110–112^ and *Pet1* has been described as the only gene whose expression is limited to hindbrain serotonergic neurons. *Pet1* precedes the expression of serotonin by 12 h and acts on the binding sites closer to genes involved in the maturation of the serotonergic phenotype. Examples include TPH2, DDC, SLC6A4 and HTR1A (5-HT1a).^111, 112^ The developmental combination of *Nkx2-2*, *Lmx1b*, and *Pet1* seem to be sufficient for the generation of serotonergic neurons in ectopic expression studies.^109^ However, across all serotonergic populations (B1–9), *Pet1* expression is not necessary for specification of ~30% of all serotonergic neurons, which have projections to highly selective targets in the brain and transcend classic anatomical subdivisions of the raphe.^51, 59, 112^

##### Subtype specification program of serotonergic neurons of raphe nuclei

Distinct subtypes of serotonergic populations (reviewed in ref. 105) have been defined in different raphe nuclei, either biochemically,^44, 48, 60, 113, 114^ based on distinct axonal trajectories and firing patterns,^115, 116^ or based on rhombomere-specific developmental programs.^48, 51, 58–61, 101, 105^ Both caudal and rostral clusters of the raphe, in the medulla and pons, respectively, contain nuclei that tend to display differential vulnerability to degeneration, but further histopathological studies would be desirable.

In the medullary raphe (caudal cluster), the raphe obscurus (ROb/B2) is more vulnerable than the raphe magnus (RMg/B3, Supplementary 1.2). The raphe obscurus (ROb/B2) is derived from R7-8 progenitors, which do not require *Egr2* expression (Fig. 4d). The raphe magnus (RMg/B3) is thought to be derived partially from R5 progenitors, requiring early developmental expression of *Egr2*,^62^ and also partially from R6-7 progenitors, which do not require *Egr2* expression (Fig. 4d). In the pons (rostral cluster), the median raphe (MnR/B8) is especially vulnerable, while the dorsal raphe (DR/B6-7) is less vulnerable (Supplementary 1.2). The median raphe (MnR/B8) originates from a mixture of R1-3 progenitors, while the dorsal raphe (DR/B6-7) originates from R1 progenitors^58^ (Fig. 4b).

Expression of *En1* and *En2* play an intrinsic role in the development of all R1-derived serotonergic neurons.^44^ Unlike other serotonergic neurons, all R1-derived serotonergic neurons require the expression of *Nkx6-1* ^51^ and do not require expression of *Insm1* for terminal differentiation.^51, 108^ In another deviation from the general specification program, a subset of R1-derived serotonergic neurons require expression of *Nkx2-2*.^51, 84^ The relative vulnerability of R1-3-derived neurons within the median raphe is not known.

#### Noradrenergic populations of the coeruleus complex

Central noradrenergic neurons are found in the medulla (A1, A2) and the pons (A4-7). Within the coeruleus complex of the pons, we focus on the LC (A6), which is the largest central noradrenergic population and the SubC (A4). Coeruleus complex noradrenergic neurons are generated from a mixture of specific rhombomere-derived neuronal progenitors located within R1-6.^63^

##### Progenitors of coeruleus complex noradrenergic neurons

During development, multipotent rostral alar progenitors are induced by FGF8 and WNT signalling, from the anteriorly adjacent isthmus organiser, enabling the expression of *En1/2*, and the AP-graded RA signalling influences all rhombomere-derived progenitor pools, which result in the expression of specific combinations of *Hox* genes (Fig. 4d and Supplementary table 1). BMPs (BMP5, 7), which are produced in the dorsal ectoderm and roof plate, establish a dorsoventral signalling gradient that specifies the identity of caudodorsal progenitors^117^ (Fig. 4d and Supplementary table 2). In mice, LC development requires NOTCH-RBPJ signalling and its direct regulation of *Ascl1* expression, as well as its indirect regulation through the target gene *Hes1*.^118^ In zebrafish, Shh signalling may play an indirect role in the maintenance of LC noradrenergic populations.^119^

##### General specification program of coeruleus complex noradrenergic neurons

In mice, the noradrenergic specification program begins with the acquisition of the correct alar progenitor identity and is followed by neurogenesis around E9 (Fig. 4d and Supplementary tables 1, 2). At least four transcription factors, *Ascl1, Phox2a/b* and *Tlx3*,^34, 120, 121^ are required for this program. Dorsal BMP signalling is required for the downstream expression of *Ascl1, Phox2a*/b^34, 117, 122^ and analysis of knock-out phenotypes for these genes suggest that they act according to a linear cascade. *Ascl1* expression is essential for dopamine beta-hydroxylase (DBH) expression in all noradrenergic populations and it induces the expression of both *Phox2a* and *Phox2b* in the LC.^120, 123–126^ *Phox2a/b* expression is required for correct specification and differentiation of LC noradrenergic populations,^124, 127–129^ and mid- to hind-brain motor neurons.^121^ *Phox2a* is required for the activation of *Phox2b*, and the latter is also necessary for the expression of DBH, which is a key enzyme in noradrenaline synthesis. Lmx1b seems to be required for noradrenergic development, since knockout mice present no vesicular monoamine transporter immunoreactivity in the brainstem.^51, 109, 130^

While *Insm1* expression is necessary for the timely onset of TH expression, the expression of *Nr2f6* ^131^ and transient expression of *Onecut1/2/3* are both required for full development of LC noradrenergic neurons.^132^ On the contrary, *Gata2* and *Gata3* are not expressed in LC and there is no strict correlation between expression of these genes and noradrenergic differentiation.^133^

##### Coeruleus complex noradrenergic neuronal subtype specification programs

Within the coeruleus complex, the LC is more vulnerable to degeneration than the SubC (Supplementary 1.3). The LC (A6) mainly originates from a dorso-alar R1 progenitor pool^63, 134^ (Fig. 4a, b). Alar progenitor pools from R1 to R6 each contribute to the SubC (A4)^63^ (Fig. 4b), which can be subdivided into dorsal and ventral parts. Like the LC, the dorsal SubC mainly derives from R1 progenitors, but can also include some R2-4 derived neurons. The ventral SubC is mainly derived from alar R4 progenitors, but can also include R2-3 derived neurons.^63^ A few R2-derived neurons are consistently observed within both the LC and the SubC, and can be identified identified by the expression of *Hoxa2*.^63^

Almost all of the LC (A6) and the dorsal part of the SubC (A4) arise from the aforementioned dorso-alar R1 progenitor pool. These progenitors require the expression of *En1* and during development they migrate ventrocaudally to a basolateral location within R1.^63, 134, 135^ A particular subset of R1-derived neurons can be further distinguished within the caudal LC as they require transient expression of *Tlx3* to induce expression of DBH.^136^ In contrast to noradrenergic LC neurons, the noradrenergic neurons of the SubC and other CNS populations (A1/2/5/7) do not require the developmental expression of *Phox2a*.^124^ Within each of these anatomically defined nuclei, especially in the C2/A2 and C1/A1 medullary nuclei, there is a subset of neurons that have not yet been associated with any particular rhombomere.^63^

#### Mesodiencephalic dopaminergic populations

Mesodiencephalic DN are organised ventrally in a continuum along the mesencephalon and diencephalon.^137–142^ In the mouse ventral midbrain, the retrorubral field (A8), SNC (A9), and VTA (A10) populations together contain 20,000–30,000 DN, representing almost 75% of all central DN.^68, 143, 144^

##### Progenitors of mesodiencephalic DN

In the mesodiencephalon, a competent pool of multipotent floor plate progenitors is generated and maintained once this region has been defined (Fig. 4d and Supplementary table 1). The correct positioning of the isthmic organiser requires *Gbx2/Otx2* ^98, 145^ and subsequent interaction between floor plate-produced SHH and isthmic-produced FGF8 is required for a ventral mesodiencephalic dopaminergic phenotype (Fig. 4d and Supplementary table 2). WNT1 is expressed in both dorsal (roof plate) and medioventral (floor and basal plate) midbrain and, like FGF8, WNT1 is also produced within the isthmus and required for the development of bilaterally flanking mesodiencephalic dopaminergic populations.^146^ TGF*β* and other members of TGF*β* superfamily^147^ are essential for the proper development of these populations.^148^ It has been described that RA signalling is involved in the terminal differentiation program where it is suggested to be essential for a SNc subset of DA neurons.^149^

Combinatorial induction diversifies genetic regulation^68^ and generates multiple heterogeneous subsets of mesodiencephalic progenitors. Along the anterioposterior axis FGF8, WNT1 and BMP are sensed differently due to variable distance to organisational centres^150^ (Fig. 4d and Supplementary tables 1, 2). At least eight different subsets of ventral mesodiencephalic progenitors have already been proposed^151^ arising from spatiotemporal inductive differences, including floor plate mediolateral differences in SHH signalling.^151, 152^ In contrast to continuous *Shh* expression within the hindbrain floor plate, mesodiencephalic precursors transiently express *Shh* due to suppression via WNT signalling, which causes a unique neurogenic response within brainstem floor plate precursors and has been suggested to be a prerequisite for differentiation of DN.^153–157^ Regional *Otx2* expression within the mesodiencephalon is essential for the unique neurogenic potential of mesodiencephalic floor plate cells^158^ (through the expression of *Lmx1a* ^68^), since hindbrain and spinal cord floor plate precursors do not appear to undergo neurogenesis.^158^ In mesodiencephalic floor plate cells, the absence of intrinsic *Otx2* expression shifts them towards a serotonergic neuronal fate.^159–161^

##### General specification program of mesodiencephalic DN

In mice, the first sign of a dopaminergic phenotype appears around E9, with the expression of *Lmx1a* and *Msx1*,^67, 150, 162^ while the corresponding mature mesodiencephalic DN are only first detectable around E10 by the expression of TH, in the absence of DBH expression.^163^ Multiple intrinsic factors and extrinsic inducers are required to activate the correct differentiation program (Fig. 4d and Supplementary table 2), which consists of many inter-dependent downstream genetic cascades.^68, 144^

The mesodiencephalic DN general specification program occurs once the corresponding progenitor markers are expressed together with *Foxa1/2, Lmx1b, Msx2* and *Neurog2*.^164^ In chick, *Foxa2* is necessary and sufficient for specification of the entire floor plate into a dopaminergic phenotype,^165, 166^ and its expression can occur via a SHH-dependent or SHH-independent pathway.^167^ In this program, both *Foxa1* ^168, 169^ and *Foxa2* ^168–171^ are necessary to promote neurogenesis by maintaining *Lmx1a* and *Lmx1b* expression,^169^ regulating the expression of *Neurog2* and *Ascl1* ^168^ and inhibiting *Nkx2-2* expression.^169^ *Ascl1* has no detected function in the development of normal mesodiencephalic DN, although it can partially rescue the generation of their precursors in the absence of *Neurog2*.

While *Lmx1a* ^172, 173^ is required for early differentiation, *Lmx1b* ^172, 174^ is an essential regulator^34^ that is co-expressed with *Lmx1a* and the transcriptional repressor *Msx1*. *Lmx1a* expression appears to be directly induced by SHH and it ultimately induces multiple proneural factors, such as *Neurog2*, and then *Msx1*.^158, 164, 175^ *Neurog2* expression starts neurogenesis, and is required for neuronal differentiation of mitotic precursors and is maintained after neuronal maturation.^150, 176, 177^ Msx1 inhibits the expression of neurogenesis regulators, such as *Nkx6-1*,^175^ nevertheless its expression is neither necessary nor sufficient for the generation of mesodiencephalic DNs.^150^ At E10.5-E11.5 in mice, both *Neurog2* and *Msx1* are responsible for proliferative cascades that allow cells to become postmitotic and to migrate radially from the initial ventricular surface into an intermediate zone of the floor plate mantle. Furthermore, loss of *Onecut1/2/3* expression results in a diminished generation of ventral mesencephalic DN.^178^

Expression of *Nr4a2* ^158, 175, 179, 180^ (*Nurr1*) is crucial for the generation and maintenance of mesodiencephalic dopaminergic populations and is downregulated in PD patients.^181^ Its expression occurs around E10.5 in mice when the corresponding mitotic precursors exit the cell cycle. This expression marks the developmental stages of both young and fully differentiated neurons, and regulates the expression of proteins involved in dopamine synthesis^182^ and transport.^183–185^ *Nr4a2* represses *Neurog2* expression and its expression can be observed across the mesencephalic flexure, diencephalon and posterior hypothalamus, although it is not exclusively present in mesodiencephalic DN.^140, 180, 182^

At later stages of development, immature postmitotic cells derived from the mesodiencephalic floor plate also express *Pitx3*,^149, 186^ which is dependent on correct regional specification by Lmx1b^187^ and modulated by En1.^69^

##### Subtype specification program of mesodiencephalic DN

In the mesodiencephalon, the SNC (A9) and the VTA (A10) are vulnerable to degeneration in PD, but SNC DN are considerably more affected than those from the VTA (Supplementary 1.4). Despite their shared origin and general specification program, subtypes of mature mesodiencephalic DN display clear phenotypic diversity.^70–73, 78^ Multiple vertebrate studies,^139, 188–193^ including in mice,^139, 194–196^ suggest that the SNC and VTA both contain mesodiencephalic DN from multiple mesodiencephalic neuromeres (M1-2 and P2-1)^68, 158, 197^ (Fig. 4a, b).

After neurogenesis and during the subsequent radial and tangential migration towards their final locations, post-mitotic mesodiencephalic cells differentiate into mature neurons. However, the developmental programs for VTA and SNC DN are distinct.^70, 78, 187^ The SNC and VTA both contain different subtypes of mesodiencephalic DN.^143, 144^ VTA DN arise from paramedian floor plate progenitors, whereas SNC DN, located lateral to the VTA, arise from median floor plate progenitors.^152, 155, 156, 198–200^

Later, during migration, the intrinsic expression of *Otx2* becomes restricted to VTA subtypes,^140, 201, 202^ while *Sox6* expression becomes restricted to SNC subtypes.^72, 202–205^

Although both SNC and VTA subtypes express *Pitx3* during terminal differentiation^143, 144^ and in aged humans,^206^ only the SNC, in terms of survival, requires expression of *Pitx3*.^68, 186, 207^ *Pitx3* supresses the intrinsic expression of *En1* and thereby of posterior markers such as CCK in SNC DN.^69, 70, 78^ Different patterns of gene expression during development permit the identification of two foetal dopaminergic neuronal subtypes that diversify into five adult mouse DN subtypes.^47, 144^ The relationship between molecular subtypes and selective vulnerability needs to be established further through advanced gene function analysis. A first sign of such a relationship has been described through the selective requirement of SNC neurons towards RA (RA) signalling and detoxification machinery in the breakdown pathway of DA. The aldehyde dehydrogenase enyme, Ahd2, depends on the activity of Pitx3 and En1, and the selective dependence of the SNC to this gene has been described.^149, 208^ This suggests that the SNC has specific requirements in term of genetic programming and metabolic demand that require the neurons to be equiped with high enough levels of Ahd2. This requirement is an inherent vulnerability in terms of function and distinguishes the SNC DN from VTA DN. Additional evidence towards this concept might be discovered through using the subset transcriptome data as described above. In addition to this, nigral neurons have a higher rate of oxidative phosphorylation and a more complex axonal arborisation.^209^ Such phenotypic differences rely on specific molecular programming as discussed above and highlight the importance of understanding the molecular machinery behind SNC programming.

## Discussion

A shared requirement for a specific set of developmental transcription factors can be used to infer functional similarity as well as proximity within a cellular developmental lineage (Fig. 4d). In PD, vulnerable neuronal populations do share certain functional similarities, so we synthesised the literature on required activity of or inactivity of a set of 51 developmental transcription factors across five brainstem regions with clear evidence of vulnerability to degeneration in PD. Within each region, we also refine our analysis to include more and less vulnerable nuclei and neuronal subtypes. Variations in the types, amount and duration of developmental induction results in different sets of required active (or inactive) developmental transcription factors, thereby shaping the landscape of lineage commitment possibilities, e.g., rhombencephalic p3 progenitors receiving a longer duration of developmental induction commit to a serotonergic fate, which correlates with an increase in Foxa2 expression and a decrease of *Nkx2-9* and *Phox2b* expression, in a switch from an otherwise motor fate.^57, 82^

Our synthesis suggests that some developmental requirements are shared between vulnerable brainstem regions. We find that vulnerable neuronal populations often share a common requirement for Shh signalling, but this induction alone is not sufficient to predict neuronal vulnerability in PD, since many other ventral brainstem nuclei do not seem to be especially vulnerable to degeneration. The activity of some transcription factors is similar in each of the five studied brainstem populations that are vulnerable to degeneration in early PD. For example, *Ascl1* is expressed in all five populations, although it is only required for the development of LC noradrenergic and raphe serotonergic populations. Together with *Phox2b*, *Ascl1* co-regulates catecholamine synthesising enzymes in noradrenergic populations.^210^ During the specification of neuronal fate, the requirement for *Ascl1* activity varies depending on the lineage of an individual cell.^211^ Absence of *Ascl1* results in loss of olfactory and autonomic neurons as well as delayed differentiation of retinal neurons.^212, 213^

*Phox2b* is required for development of noradrenergic neurons, and visceromotor neurons of the dorsal motor nucleus of the vagus (10N), while its paralogue *Phox2a* is also required for LC, but not subcoeruleus (SubC) noradrenergic neurons. Trochlear motor and oculomotor neurons share the same *Phox2a* and *Phox2b* developmental requirements as LC noradrenergic neurons,^121, 128^ however these neurons do not seem to be vulnerable in PD. In most of the brainstem, *Phox2b* represses serotonergic differentiation and therefore it is required to be absent for specification of raphe serotonergic neurons. Although *Phox2b* is expressed in caudal midbrain dopaminergic populations, it does not seem to be required for the specification of substantia nigra, pars compacta (SNC) DN.^214^

In mice, *Lmx1b* expression is required for the expression of monoamine vesicular transporters in all brainstem aminergic neurons (dopaminergic, noradrenergic and serotonergic),^51, 109, 130, 174^ despite not being required for zebrafish LC noradrenergic populations.^184^ Visceromotor neurons of the 10N do not express vesicular monoamine transporters,^33^ but it is not known if *Lmx1b* is required for the development of these neurons. Importantly, *Lmx1a/b* is required to control autophagic-lysosomal function, integrity of nerve terminals, long-term survival of midbrain DN^215^ and recently has been implicated in regulation of mitochondrial function.^216^ *Lmx1a/b* conditional ablation, after neuronal specification, results in abnormalities that show striking resemblance to early cellular abnormalities seen in PD. Moreover, a decrease in *Lmx1b* expression has been reported in midbrain DN of PD patients.^215^ Finally, it has been shown that aspecific subset of SNc neurons is absent in Lmx1a mutants.^217^

Within each of the five vulnerable brainstem regions we considered, different nuclei are more or less vulnerable to degeneration in PD. Of the visceromotor neurons, those in the 10N are more vulnerable to degeneration than those in the inferior salivatory (IS) nucleus.^7, 11^ Even though these nuclei share the same general specification program, the IS originates from the R6 rhombomere, while the 10N originates from the R7-8 rhombomeres.^64, 65^

Differential vulnerability within mesodiencephalic dopaminergic nuclei is well established and there also exist differences in their developmental specification programs. Medial and paramedial mesodiencephalic floor plate progenitors generate DN in the SNC and VTA, respectively. Both express *Pitx3*,^143, 144, 206^ but knock-out of *Pitx3* results in selective loss of SNC neurons,^68, 186, 207^ so *Pitx3* expression is required for the development of the SNC but not required for the VTA.

Within some vulnerable nuclei, the existence of different developmental programs that generate different neuronal subtypes are known, e.g., three dopaminergic neuronal subtypes can be distinguished in human VTA.^144^ Within other vulnerable nuclei, differential vulnerability of anatomically distinct areas is known in PD, e.g., there is an increasing fraction of cell loss from medial to dorsolateral SNC.^218^ However, to our knowledge, a simultaneous analysis of developmental subtype-specific markers and anatomically resolved quantification of cell loss in PD have not been reported. We suggest that tissue samples from previous cell loss studies should be immunohistopathologically revisited to check if there is a relationship between the relative degree of neuroprotection of neuronal subtypes that are defined by differential expression of known developmental transcription factors responsible for adult maintenance.

The developmental origins of selectively vulnerable neurons needs further clarification. Further experimental work is required to assess the temporal requirement for certain transcription factors (e.g., *Onecut, Gata2/3, Isl1, Insm1, Pet, Tbx20, Nr2f6, Nr4a2, Pitx3*, Lmx1a) that seem to be necessary for specification of a subset of nuclei but are not yet known to be required for other vulnerable nuclei that we have considered. The need for data on the timing of requirements is supported by evidence that *Nr4a2* is required for maintenance of mesencephalic DN in adult mice.^219^

This review presents a comprehensive manual curation of the development of ten vulnerable brainstem nuclei in five different brainstem regions. Our compendium of transcription factor requirements is accurate but not yet comprehensive at genome scale. This limitation can be partially overcome by complementing our compendium with developmental omics data, e.g., the Allen developmental primate atlas.^220^ In turn, the noise in such large scale datasets can be mitigated by using our manually curated transcription factor requirements as an anchor to benchmark data integration algorithms. Ultimately, cell fate mapping and gene inactivation studies are required to establish the combination of genes required for developmental specification of each neuronal subtype.

## Conclusions

In early PD, mature neurons that are selectively vulnerable to degeneration can be identified by some shared biochemical, morphological and functional characteristics. However, the molecular basis for selective vulnerability in PD remains to be fully elucidated. As mature neuronal identity is largely the result of a developmental program that is specific to each cell type (Fig. 4e), for five brainstem regions, each with at least two nuclei with varying degrees of vulnerability, we compared and contrasted their cellular lineage and their requirement for absence or presence of 51 transcription factors (Fig. 4d). Certain combinations of transcription factors seem to be required for development of many vulnerable brainstem regions, e.g., *Ascl1* coregulates catecholamine-synthesising enzymes in noradrenergic populations.

Within vulnerable brainstem regions, certain nuclei are more vulnerable to degeneration than others and this correlates with important differences in the developmental transcription factor requirements for their lineage, e.g., *Pitx3* is expressed in all mesodiencephalic DN but it is only required for development of SNC, but not ventral tegmental area, DN. Of the vulnerable visceromotor neurons, those from the inferior salivary nucleus are less vulnerable than those from the dorsal motor nucleus of the vagus, yet they both have almost the same developmental program, except that they originate from separate rhombencephalic neuromeres. Tracing the molecular consequences of developmental specification programs in more and less vulnerable brainstem nuclei, e.g., with experimental determination of the genomic targets of key transcription factors would help to identify the molecular species that participate in the biochemical pathways that could be associated with differential vulnerability. The development of a comprehensive molecular basis for the shared characteristics of vulnerable neurons is an essential pre-requisite for development of drugs targeted towards the causes of PD.

## Methods

To completely reconstruct an anatomically resolved cellular developmental lineage of adult human neurons as well as the corresponding developmental transcription factors would require human experimental data, which is not available. Fortunately, the brainstem and its development is highly homologous between mammalian species. It also contains the most archaic neuronal networks in the brain, which may be related to susceptibility degeneration in PD.^221^ Therefore, we relied on manual curation of developmental studies in model organisms to obtain the details of neuronal progenitor patterning, neurogenesis and cell fate specification^222, 223^ as well as the details on genoarchitecture and neuromere-related lineage mapping.^224^ Unless indicated otherwise, all statements refer to murine studies. Differentiation and fate restriction requirements were studied by considering multiple loss-of-function studies, which describe the dependence of each neuronal population on a specific set of gene products. In this regard, when possible, we highlight whether a particular gene is necessary to be active, or necessary to be inactive, for each lineage (Fig. 4d). Furthermore, migration patterns were selectively reconstructed by curating lineage tracing studies (Fig. 4c). We used the same neuromere scheme and ontology as the Allen Developing Mouse Brain reference atlas.^142^

### Data availability

The authors declare that the data supporting the findings of this study are available within the paper and its supplementary information files.

## Electronic supplementary material

Supplementary Information
