# Supplementary material for: Embryonic development of selectively vulnerable neurons in Parkinson’s disease
Source: NPJ Parkinsons Dis. 2017 Jun 26;3:21. doi: 10.1038/s41531-017-0022-4 (PMC5484687; doi:10.1038/s41531-017-0022-4)
Supplement: Supplementary file 1 — Supplementary Information [file 41531_2017_22_MOESM1_ESM.pdf]

Supplementary Information

1 Characterisation of vulnerable neuronal populations

1.1 Mature visceromotor neurons of the dorsal motor nucleus of the vagus

Degeneration: The 10N was one of the first nuclei to be implicated in PD, when Fritz Jakob Lewy identified intracellular protein aggregates within this nucleus, while analysing brain samples from post-mortem PD patients.[1] For virtually all patients, a strong Lewy pathology was reported within the population of parasympathetic preganglionic neurons which project their axons via the vagus nerve and that apparently lack neuromelanin.[1,2]

Cell loss experiments have also shown a reduced number of cells within the 10N. Nevertheless, there is still a debate about the most vulnerable neuronal populations. Some studies claim loss of neuromelanin-containing neurons, while others have not been able to confirm this.[1] It has been suggested that the preganglionic parasympathetic projection neurons are the first to degenerate[3] and that cell loss may only occur at a later stage of the disease.[1]

Location and biochemical characterisation: The centrointermediate subnucleus (10Cel) of the 10N holds the visceromotor preganglionic component of the vagus nerve, which exits the brainstem dorsolaterally and is claimed to be the most susceptible 10N neuronal population in PD.[4] This subnucleus is one of the eight different subregions that have already been identified within the 10N and also one of the first to develop in the human brain, together with the dorso- (10DI) and the ventro-intermediate (VI) subnuclei.[5,6]

In rats, visceromotor neurons of the 10N (from 10Cel) are mainly generated around E10-11 and have been suggested to be cholinergic, since they express choline O-acetyltransferase (CHAT).[7,8] These CHAT-positive cranial motor neurons are located dorsally or dorsolaterally to the hypoglossal nucleus.[7] In mice, the cell bodies of these neurons form a column within the central portion of the medulla within rhombomere R7 and R8.[9,10] The arrangement of all cranial motor neurons within the CNS and their corresponding innervations show a high degree of conservation between vertebrates.[10] In the Allen Human Brain Reference atlas, the 10N is part of the efferent nuclei of cranial nerves in the medulla oblongata (MoEN).

These visceromotor neurons express calbindin[5] and the catecholaminergic enzymes tyrosine hydroxylase (TH)[5] and aromatic acid decarboxylase (DDC). Despite the expression of these enzymes, which imply the existence of cytosolic catecholamines, these catecholamines may not be released due to the lack of vesicular monoamine transporters.[1,11] Several other human post-mortem studies have characterised these neuronal populations and discovered high concentrations of receptors for somatostatin, cannabinoids and dopamine (D2 and D4), together with dopamine beta-hydroxylase (DBH) and bombesin.[12] The 10N also holds a noradrenergic group of neurons, called the A2 group.[1]

Function and phenotype: Cranial motor neurons can generally be classified according to three subsets, the visceral motor (VM), the branchiomotor (BM) and the somatic motor (SM) neurons, whilst the spinal motor neurons only include SM and VM neurons.[9] The 10N visceromotor populations are preganglionic (efferent) neurons[13] that belong to the cranial subset and represent the largest parasympathetic population in the brainstem. Their axons project via the vagus nerve and navigate long distances from the CNS towards the correspondent peripheral parasympathetic ganglia[9,10] and synapse directly with a subset of neurons within the myenteric and submucosal ganglia.[14] These motor fibres represent just a small portion of the existing fibres in the vagus nerve, which mainly contain afferent sensory fibres,[13] and their axonal terminals form a characteristic network of fibres arborising around the target ganglia.[14] 10N visceromotor neurons are key regulators of autonomic functions within the viscera, the thorax (heart, lungs, esophagus) and the abdomen (spleen, pancreas, small intestine, proximal large intestine).

Dysfunction of cranial motor neurons is frequently associated with specific symptoms, which are characteristic of some neurodegenerative disorders, such as PD and amyotrophic lateral sclerosis. In the latter, there is a progressive loss of the upper motor (corticomotor) and lower motor (brainstem and spinal cord) functions. Therefore, possible correlation between the degeneration of the cranial motor neurons in the aetiology of amyotrophic lateral sclerosis can also be taken into consideration when studying PD.[15]

1.2 Mature serotonergic neurons of the lower and upper raphe nuclei

Degeneration: Most severely affected are serotonergic populations of the median raphe (MnR/B8)[16] and of the raphe obscurus (ROb/B2),[16] located in rostral and caudal clusters of the raphe nucleus, respectively. In PD patients, the median raphe population (MnR/B8) can be reduced by one-half[16,17] and presents with some evidence of Lewy pathology.[1,16] On the contrary, the raphe obscurus (ROb/B2) presents a comparatively smaller fraction of cell loss,[16] but with greater evidence of Lewy pathology within medium-sized lipofuscin-laden neurons.[1,2,4,16] Other raphe populations have also been associated with PD, due to the presence of either Lewy pathology and/or cell death, although the relation has not yet been fully described.[4] Examples include the raphe magnus (RMg/B3) and the dorsal raphe nucleus (DR/B6-7).

Location and biochemical characterisation: Differential gene expression studies have suggested an homeodomain code that distinguishes these two clusters during development.[18,19] Indeed, recent transcriptomic datasets have provided new markers to enable the classification of different sub-types of serotonergic neurons within raphe nuclei.[18] However, only a few markers are able to distinguish the molecular differences between these populations.[18] Gene expression profiles of heterogeneous rostrocaudal sub-types of raphe serotonergic neurons are complex despite their common monoaminergic character. All serotonergic populations in raphe nuclei are identifiable by the enzymes involved in the synthesis of serotonin, including tryptophan hydroxylase (TPH2) and L-aromatic amino acid decarboxylase (DDC). Moreover, they are also characterised by the expression of the serotonin transporter, SLC6A4.

Function and phenotype: Raphe nuclei contain different groups of central serotonergic neuronal populations (B1-B9), intermingled in variable portions with other non-serotonergic neuronal populations.[4] Their serotonergic populations are phenotypically different, but share a common predominance of medium-sized, multipolar projection neurons that contain large quantities of lipofuscin pigment granules.[4]

Raphe nuclei are located throughout the hindbrain and are classically subdivided into a rostral and a caudal cluster. The rostral cluster is named raphe pontis (PnRa/B5-9) or upper raphe and is located in the pons. The caudal cluster is named raphe nuclei in the medulla oblongata (MoRa/B1-4) or lower raphe. Each of these clusters contains different nuclei that project to different locations in the brain. Different raphe populations show variable degrees of Lewy pathology and cell death in PD. The MoRa predominantly contains descending projections and consists of the raphe magnus (RMg/B3), the raphe pallidus (RPa/B1) and the raphe obscurus (ROb/B2). These descending axonal projections modulate a diverse set of physiological processes, such as cardiorespiratory homeostasis, thermoregulation, and nociception.[20–24] In turn, the PnRa contains the somata of the ascending projections of several nuclei, including the dorsal raphe (DR/B6-7), the median raphe (MnR/B8), the paramedian raphe (PMnR/B9) and the raphe pontis nucleus (PRn/B5). These ascending pathways are responsible for the modulation of emotional responses, circadian rhythms and energy balance.[25,26]

The serotonergic system has a modulatory function and its very diffuse innervation results in a very broad direct and indirect induction of widespread brain targets. Therefore, serotonergic neurotransmission has been implicated in the regulation of a vast array of neuronal circuits of

behavioural and physiological states and functions, such as sleep, mood, appetite, anxiety, and neurovegetative control.[27]

Some genetic perturbations of developmental and specification processes have been linked with alterations in rodent behaviour[24,28,29] and are thought to significantly contribute to susceptibility of human emotional and stress-related neurodevelopmental pathogenesis in adulthood[30].[18] Thus, a correct expression of developmental transcription factors is crucial for the correct specification of all raphe populations. Some polymorphisms within the correspondent genes have been correlated with functional changes in their protein levels or biological activities.[31] Genetic studies have further highlighted these polymorphisms (in single or combined nucleotides) as predisposition factors for several neuropsychiatric disorders, such as anxiety and depression.[32–35] In this regard, it's important to understand how the phenotype of the different raphe neuronal subsets is acquired and regulated during development.[36]

1.3 Mature noradrenergic neurons of the LC/SubC

Degeneration: The LC nucleus has been identified as severely affected in virtually all PD patients. The affected populations are noradrenergic neurons from the A6 group, located within the central portion of the LC and projecting to the temporal cortex and hippocampus.[16] Lewy pathology has mainly been observed in large projection neurons that contain neuromelanin[2,4,37] and is not observed in non-melanin-containing neurons.[1,16]

Many other studies report cell loss within the coeruleus and subcoeruleus complex, in both noradrenergic[4,16] and neighbouring populations within the LC.[38] Despite these findings, there is an inconsistent correlation between cell loss and age in these nuclei.[39]

Location and biochemical characterisation: LC neurons are among the earliest-born neurons in the brain and can be found in the R1 from E9.5 onward,[40–46] together with their radial migration towards the pial surface. In subsequent steps, around E14.5, these populations migrate ventrocaudally and settle in the lateral basal plate, close to the forth ventricle.[45–47]

The coeruleus-subcoeruleus complex consists of the LC and subcoeruleus (SLC) nucleus, containing the A6 and A7 groups of noradrenergic neurons, respectively.[4] In humans, both populations locate in the dorsal part of the pons, within the pontine tegmentum, and in mouse the A6 group is located within the rhombomere R1, while the A7 group is located in both rhombomeres R1 and R2.[48] Most of these neurons are pigmented and secrete the neurotransmitter noradrenaline.[39] This population is also known to express α2-adrenoceptors, which are suggested to decline in number with age.[39]

Function and phenotype: The LC A6 neuronal population is the major noradrenergic centre in vertebrate CNS,[44] with a widespread network of projections that contribute to the regulation of arousal, attention, sleep/awareness, and adaptive behaviour.[46] Phenotypically, these neurons have long, thin, and sparsely myelinated axons that project to the striatum, cerebellum, and multiple other areas in the cortex and portions in the lower medulla.[4,39]

1.4 Mature dopaminergic neurons of SNC and PaN

Degeneration: In mid-stage PD patients, there is a heterogeneous loss of DN within substantia nigra populations and the most affected neurons reside within the SNC (A9).[16,49,50] This loss appears to be specific to PD[51] and its progressive degeneration is the best documented lesion.[1] One of the latest studies addressing degeneration in the SNC estimated that one-third of the striatal dopaminergic terminals are affected.[52] Nevertheless, this estimation is low in comparison with the of reported 50-80% reduction, which is generally considered to be with the appearance of motor

symptoms.[4] It is also important to note that substantia nigra degeneration and motor dysfunction generally correlate in PD, although in some other diseases with parkinsonian symptoms, these neurons do not exhibit cell loss.[53]

Lewy pathology appears in melanised neuronal populations of SNC posterolateral subnucleus[2,4] and progressively affects other posterior subnuclei (posteromedial and posterosuperior).[54,55] Several other immunocytochemical studies have also confirmed that neurons located in a more ventral tier are more vulnerable than those in a medial position.[56] Furthermore, SNC DN are more vulnerable to cell death than the ones of other nuclei, which lie around the same ventral location.[57–60] Melanised dopaminergic populations of the PaN (A10) and PBP (A10) display less pronounced changes in PD patients. Moreover, ventral tegmental area populations (VTA/A10) are relatively spared from disease pathology.[61] Unfortunately, there is still no consensus as to the reason for this apparent resistance to degeneration.[4]

Various molecular[62] and electrophysiological[63] studies have already highlighted the existence of an intricately organised complex of interdigitated dopaminergic neuron sub-types within mesodiencephalic dopaminergic populations.[64,65] Generally located in similar but distinct anatomical locations, these neurons apparently have different sensitivity do degeneration, connectivities and functional characteristics.[66,67] These differences highlight some distinct morphological features, co-transmitters and other distinctive marker proteins.[68]

Biochemical characterisation: In the SNC, ventral tier neuromelanin-containing neurons are specifically susceptible to degeneration,[61] although some of the most highly pigmented neurons in the brain are spared in PD.[69,70] These populations present low levels of calbindin (CALB1)[55,71] and SLC18A2, higher levels of SLC6A3, as well as decreased vesicular accumulation of dopamine.[1,54,65,72,73] They are also immunopositive to the potassium channel KCNJ6 (GIRK2), to D2 dopamine receptors and to lactotransferrin (LTF).[56]

Function, phenotype and location: The substantia nigra is located in the inferior midbrain tegmentum and extends from the posterior tip of the mammillary body to the oculomotor nucleus.[74] This nucleus holds three different zones, including a cell-dense pars compacta (A9), a cell-sparse pars diffusa and a reticulate portion. Additionally, within the pars compacta portion, seven other subnuclei can be distinguished, according to their location.[75]

SNC DN are located close to the floor plate, at the ventral rim of the neuroepithelium,[76] and have a spherical and marginally located nucleus, with neuromelanin granules at the other cell pole. These neurons have a few thick dendrites that are arranged in bundles and do not generally project beyond the boundaries of the substantia nigra. Moreover, their axons project to the dorsal striatum via the nigrostriatal pathway. These axons are of a fine-caliber, are thinly myelinated and have a thick cone-shaped initial segment.[4] These populations are required for the control of voluntary movement and their loss results in an impairment of motor function.[68,77]

In humans, the A10 group consists of seven nuclei, which include the parabrachial pigmented nucleus, the PaN and the ventral tegmental area.[78] The PaN (A10) and the PBP (A10) are located in the ventromedial midbrain tegmentum and provide the major afferent dopaminergic projections to the amygdala, the hippocampal formation, and the entorhinal region.[4] Moreover, the dense dopaminergic projections from the PaN reach the neocortical motor areas, the anterior cingulate fields and the prefrontal association areas.[4] Likewise, the ventral tegmental area (VTA) is located in the ventral midbrain tegmentum and its neurons innervate the ventral striatum and the prefrontal cortex via the mesocorticolimbic system, which is involved in the regulation of emotion and reward.

2 Neuronal and neurotransmitter identity of brainstem monoaminergic populations

Phenotypic characterisation of neuronal morphology and electrophysiology are very informative but do not provide as many measurable attributes as, for example, transcriptomic data.[79] Neurotransmitter and electrophysiological phenotypes can be correlated with the transcriptomic signatures of mature neurons, but such correlation is more challenging for quantitative morphology and connectivity. Also, morphology and connectivity is determined by transcriptional programs that are only active during development.[80] In contrast, enzymes involved in neurotransmitter biosynthesis as well as the corresponding receptors and ion channels are required to be expressed in a mature state and form part of the mature neuronal signature.[79]

The developmental choice of neurotransmitter is one of the better documented aspects of neuronal identity (Figure 3) for which many key regulatory genes and crucial extrinsic factors have already been revealed.[44] Most of the vulnerable populations in PD produce monoamines that can be used for neurotransmission, which confers a similar neurotransmitter identity defined by the expression of similar key biosynthetic enzymes[44](Supplementary information 1). These biogenic neurotransmitters are synthesised from aromatic amino acids, such as phenylalanine, tyrosine or tryptophan and can be classified as cathecholamines (dopamine, noradrenaline and adrenaline), tryptamines (serotonin and melatonin), tyramines, octopamines and histamines.[81]

Monoaminergic neurons are broadly distributed and classified according to their position, where the A group represents the catecholaminergic populations and the B groups the serotonergic populations.[81] The mouse dopaminergic populations are classically grouped from A8 to A17, where the mesodiencephalic population A9 corresponds to the SNC and the A10 population to the ventral tegmental area and the PaN[81] (A15 is part of the olfactory tubercle and A16 is part of the olfactory bulb[81]). Noradrenergic populations are represented in the medullary A1 and A2 groups and in the pontine A5 to A7 groups, where the A6 population forms part of the LC.[81] All mouse serotonergic populations are grouped from B1 to B9 and originate from the hindbrain, the medullary B1 to B4 and the pontine B5 to B9 . The raphe nuclei include most of these populations.

Within PD vulnerable populations, the A9 group correspond to DN of the SNC, the A6 correspond to the noradrenergic populations of the LC and both B5-9 and B1-4 correspond respectively to the serotonergic populations of PnRa and MoRa. Cholinergic 10N populations apparently do not use a monoamine as neurotransmitters, but they also share some key biosynthetic enzymes, such as TH, and are able to store a monoamines. Each type of monoaminergic neuron expresses specific set of enzymes and transporters involved in neurotransmitter synthesis, packaging into vesicles and re-uptake into the neurons after release. The coordinated co-expression of these enzymes can be depicted by the analysis of the transcription factors involved in their terminal differentiation.[81]

3 Summary of brainstem development

3.1 Embryonic developmental steps

The CNS develops from a common population of intra-embryonic stem cells originating from the inner cell mass of the blastocyst (13-cell inner cell mass). In an early embryological event (E3.5 mouse and E5 human[50]), this inner cell mass population starts to divide and to change its physical position to form an inner bi-laminar disk (the embryo itself) located internally in the blastocyst. This bi-laminar disk consists of two cell mass layers, a dorsal epiblast layer that lines the anionic cavity and a ventral hypoblast layer that lines the primary yolk sac.

3.1.1 Gastrulation

In a process called gastrulation, the embryonic bi-laminar disk will turn into a tri-laminar disk at the medial anterior portion of the epiblast layer (anterior primitive streak). This process starts with

FGF8 induction, which is responsible for the down-regulation of e-cadherin (that binds the epiblasts together) and up-regulation of brachyury (essential transcription factor in mesodermal formation). This change of expression allows these epiblasts to migrate into deeper ventral layers of the embryo through the primitive groove, which is the result of the primitive streak depression.

The primitive streak plays an important role in the development of the body, since it allows space for the migration of anterior epiblasts. The first population to migrate will form the intra-embryonic endoderm which is displaced next to the hypoblast layer, whereas the second population will form the intra-embryonic mesoderm which positions in an intermediate zone. At the same time, the epiblasts from the dorsal germ layer, which does not migrate, will generate the intra-embryonic ectoderm. The hypoblast layer will become the extra-embryonic endoderm. This three-layered structure is called the gastrula and its intra-embryonic ectoderm will eventually form the skin and neural tissues. The intra-embryonic mesoderm will form muscles and bones, while the intra-embryonic endoderm will form the cells lining the digestive and respiratory tracts.

These embryological steps are reviewed elsewhere,[50] but it is safe to assume that the differentiation of both mouse and human embryonic stem cells follow a similar set of hierarchical signals. Therefore, they both share a similar regulation of the embryonic development with further generation of the different germ layers and specific cell types.[50]

3.1.2 Neurulation

The development of the entire nervous system begins during gastrulation by a process called neurulation and results in the formation of the neural tube. During neurulation, stem cell populations from the dorsal midline of the intra-embryonic ectoderm start to generate two other populations of specialised neuroectoderms: the neural plate ectoderm and the neural plate border cells. The former is the precursor for the entire CNS, while the latter forms the PNS by differentiation into neural crest populations.[82]

The neural plate populations are first generated and differentiated in the anterior/cranial end of the embryo (cranial neurulation) and then proceed in a posterior/caudal direction[83] (spinal neurulation).[84] Neurulation lasts from the appearance of the neural plate to the closure of the neural tube. Both steps of neurulation have different mechanisms and timings along the anteroposterior axis which distinguish them as cranial or spinal neural plate.[83,84] Therefore, cranial neural plate cells are a specialised anterior neural plate ectoderm, destined to undergo neurulation and to generate the cranial portion of the neural tube, whose progression is different from that seen in the spinal region.[82] A considerable portion of this cranial neural plate is ventrally underlined by the notochord and corresponds to the prospective brainstem.

Neural tube convergent extension In mammals, the formation of the cranial neural tube begins with the bending of the cranial neural plate. This event occurs at the Medial Hinge Point (MHP), overlying the notochord, and at the Dorsal Lateral Hinge Point (DLHP), located at the attachment point of the surface ectoderm to the outside of each Neural Fold.[85] After the biconvex Neural Folds are formed, they bulge outwards due to cranial mesenchyme-marked expansion. Then, they flip around and approach until they meet at the dorsal midline (neural tube closure).[82,86] These movements are known as convergent extension and depend on highly conserved WNT-frizzled signal transduction pathways.[82,87] In mouse, the primary neural tube closure initiates at the hindbrain/cervical boundary on embryonic day E8.5, equivalent to a approximately 3 weeks post fertilisation in humans.[84] Before the complete closure of the neural tube, neuronal induction becomes determinant due to the anterior-posterior (AP) and dorsal-ventral (DV) patterning events that start the regionalisation of this structure.

During early embryonic stages, the populations of embryonic stem cells face constant changes of their physical location, but remain pluripotent and mitotic until the beginning of neuronal induction. The neural plate ectoderm is entirely proliferative and after neuronal induction, the cells start to exit the cell cycle and to commit to a neuronal differentiation program. Differentiation only occurs after

complete closure of the neural tube for each position in the body axis.[82] Notch signalling determines the timing of the balance between proliferation and differentiation in cranial neurulation.[82]

Although embryonic stem cells retain their stem cell characteristics until neuronal induction, throughout the earlier stages of development they gradually start to present some degree of commitment. These changes mainly occur in the length of some of the cell cycle stages, mainly G1 and G2, which results in diminished pluripotency.

The correlation between the timing of the neural tube closure in mouse and human, at different anterior-posterior positions, is still under debate, meaning that they may not share exactly the same exact order of events.[84] Therefore, this is an important issue to consider in the study of developmental lineages, because it may imply slightly different neurulation events between mouse and human. Differences in such an important developmental step between mouse and human, like it is neurulation, may be represented by slightly different induction mechanisms, which downstream may reflect important differences in gene expression profiles of the generated populations.

In the current work, we give special attention to the developmental lineage of specific brainstem populations that are originated from the anteriorly positioned non-migrated epiblasts. These epiblasts generate the anterior neural ectoderm, the subsequent cranial neural plate and the cranial neural tube. Only the populations in the cranial neural tube, which are underlined by the notochord, are considered in this study, since they are the mitotic precursors of all brainstem populations.

3.2 Brainstem patterning and neural induction

Most of the classical studies on vertebrate neuronal patterning were performed on embryos of birds and amphibians.[88] However, over the last two decades, fish and mouse models have made an important contribution. Neuronal induction specifies the identity of different progenitors and specific inductive combinations are required for the generation of all neuronal subsets in the CNS. Therefore, the identification of the source and the nature of these early inductive signals, together with their embryological, cellular, and molecular basis will help to understand the individual fate restriction programs that occur in each individual developmental lineage.[50] Neuronal fate is controlled by a combination of intrinsic factors and extracellular signalling molecules that act as positive or negative regulators of neuronal differentiation.[89–92] Positive regulators such as sonic hedgehog (SHH) promote the commitment and differentiation of neural stem cells, while negative regulators such as NOTCH,[93,94] EGF and FGF2[95,96] maintain the self-renewal and the multipotent status of undifferentiated neural stem cells.

During gastrulation, the action of inductive signals begins the specification of the dorsal ectodermis into a neuronal fate. This initial induction leads to the formation of the neural tube, which starts to show evident differences in shape along the AP axis, and differences in gene expression (mostly transcription factors) across both AP and DV axes. This induction empowers the neuronal precursors with the ability to produce a great diversity of region-specific progenitors and differentiated populations. The variety of possible combinatorial inductions generate distinct identities, in terms of morphology, axonal trajectory, synaptic specificity, neurotransmitter content and other characteristics.[50] In the prospective brainstem, neuronal induction of embryonic stem cell precursors begins with the first neurulation and occurs concomitantly with neuronal patterning. Neuronal patterning is one of the most studied neuronal inductions and its action relies on two important aspects, a spatial and a temporal specification.[50,97]

In the spatial specification, morphogens generated and secreted in specific locations, within the neuroepithelium and surrounding tissues (by organisational centres), signal and pattern the AP, DV and right-left (RL) axes of the CNS.[50] Mixtures of gradients of developmental morphogens throughout the neural tube generate important positional information (regionalisation), which can be represented as a molecular grid of neuronal induction.[98] The initial position of a precursor defines its exposure to morphogens that progressively restrict their developmental potential.[99]

Cell fate is directed by the activation or repression of transcriptional regulators, which control the genetic network necessary for the generation of the specific phenotype of each neuronal cell type.[99]

Initially, these environmental cues induce the generation of mitotic progenitors with multiple gene expression profiles, which later will generate mature populations. Understanding the mechanism by which extracellular concentrations of morphogens are translated into the intracellular specification is vital to address lineages development.[100] Morphogens act at a distance from their source point, directly on the recipient cells without any relay mechanisms in a concentration-dependent manner.[100] Some of these morphogenes include bone morphogenetic proteins (BMP), fibroblast growth factors (FGF), retinoids, the NOGGIN, SHH[88] and the wingless-type protein (WNT).

Temporal specification governs the order and hierarchy of cell fate decisions,[50] thus, for each specific location in the neural tube, cells are generated on a precise and predictable temporal schedule, with sub-types of neurons appearing in a defined order.[97] The timing of cell generation can be encoded within the early progenitors as a cell-intrinsic program and the extrinsic signals from the morphogens are important modulators of this program.[97] CNS progenitors undergo a series of asymmetric cell divisions to generate the first neurons and their lineage trees in mouse are remarkably similar to those in invertebrates.[97,98,101,102]

Patterning events are the processes by which embryonic cells form ordered spatial arrangements within tissues. They occur concomitantly with neuronal induction and establish the specific profiles of transcription factor expression within neuronal progenitors, giving rise to distinct classes of post-mitotic cells (reviewed elsewhere[103,104]). Each of these individual profiles define a specific progenitor domain by orchestrating key neuronal aspects, like cell-specific programs that define the migration, projection pattern, and synaptic specificity of neuronal sub-types.[105]

A complete understanding of how cells interpret and consequently generate a global response to each of the extracellular signals remains an unmet challenge. A first hypothesis is that each signalling pathway triggers a specific transcriptional signature and the sum of these signatures would indicate a neuronal progenitor identity to adopt. However, several evidences point to more direct connections between the different intracellular signalling cascades activated by these inducers. For example, SHH and WNT pathways seem to interact physically and also *Gli3* expression (*Shh* effector) is regulated and dependent on the WNT activity.[105]

3.2.1 Anterior-posterior patterning and neuronal induction:

The AP patterning of vertebrate CNS begins early in development, during gastrulation, starting the CNS regionalisation. By embryonic day E9.5 in mouse, the main regions of the CNS can already be distinguished morphologically along this axis.[106] The anterior neuronal ridge and the isthmus organiser are two important organisational centres implicated in AP patterning of the caudal forebrain, the midbrain and the anterior hindbrain, through the secretion of the morphogen FGF8. Also, all hindbrain is patterned by a combinatorial expression of Hox genes that are induced by retinoic acid.

Prosomeric model The anterior-posterior patterning events organise the brainstem in a series of transverse units called neuromeres, which are comprehensively described by the prosomeric model. According to this model, the mapping of each neuromere is relatively straightforward and reproducible due to the existence of multiple neuromeric landmarks. Each neuromere is named differently according to its location and in the Allen Developing Mouse Brain Atlas,[48] the brainstem consists of: 3 prosomeres (P1-3), which divide the diencephalon,[107] 2 mesomeres (M1-2) which divide the mesencephalon and 12 rhombomeres (is-R11) which divide the rhombencephalon. Note that there are authors who consider an alternative classification for some of these neuromeres, where the isthmus (or rhombomere "R0") and "R1" can be grouped within an extra-large "R1" domain and all rhombomeres from "R8" to "R11" fit into a large "R8" rhombomere.[19]

The mesodiencephalic neuromeres (P3-1 and M1-2) share similar anterior-posterior patterning events, which result in the development of common trans-neuromeric mature populations. In the development of the hindbrain, the transiently segmented rhombomeres (is-R11) will generate the pons,

medulla, and cerebellum. In mice, these transverse units can be grouped in to 4 main locations (Allen Developing Mouse Brain Atlas[48]): the anteriorly positioned prepontine (is-R2), the intermediate pontine hindbrain (R3-R4), the pontomedullary hindbrain (R5-R6) and the posterior medullary hindbrain (R7-R11). In the posterior medullary hindbrain, these rhombomeres are generally called crypto-rhombomeres (cR8-11), since the inter-neuromeric limits have not yet been described in mouse as morphologically distinguishable partitions.[19] These crypto-rhombomeres were first described in chicks (as pseudo-rhombomeres) via experimental fate-mapping studies[108] and latter corroborated by the specific expression of *Hox* genes.[109] In mouse there are reasons to assume the same hidden partitions.[19,110]

In the hindbrain, the various rhombomeric partitions have specific expression patterns of *Hox* homedomain genes (*Hox code*) and other specific molecular determinants that support their rhombomeric segmentation.[109,111] In this regard, extensive studies have unravelled the different Hox gene expressions in each rhombomere, which specify their different anterior-posterior identities. Alterations in these Hox genes can cause homeotic transformations of the rhombomeres and also affect the specification of discrete neuron types.[112] Also, simply looking into the rhombomeric topography and other variations occurring in development, it is also possible to identify some peculiar differences between these units. For example, paired rhombomeres are normally advanced in neurogenesis relative to the unpaired ones.[19] This development with a two-segment periodicity ensures the expression of differential cell adhesion profiles preventing the intermix of cells between compartments, despite the non-existence of physical barriers between rhombomeres.[105,113]

Anterior-posterior patterning events are responsible for brainstem segmentation during development and each neuromere must have a specific developmental program, which enables the production of specific neuronal types, such as the catecolaminergic, the visceromotor and/or the brachiomotor neurons, as well as other anatomical derivatives.[19]

Isthmus organiser - FGF8 and WNT1 signalling The isthmus organiser is located within the junction of the future midbrain and the anterior hindbrain[114–116] and plays a determinant role in neuronal induction of these surrounding areas. It acts through the secretion and diffusion of the morphogenes FGF8 and WNT1, which signal different inductive cascades within neuronal precursors.[106] The development of this organisational centre requires the expression of the genes *Otx2, Gbx2, Pax2, Lmx1b, Wnt1, En1* and *En2*.[68]

Downstream to this induction, a set of specific developmental transcription factors start to be expressed. In mice, as early as embryonic stage E7.5 it is already possible to distinguish two differently specified regions in the neural plate: one anteriorly located expressing *Otx2* and another posteriorly located expressing *Gbx2*.[106,117] At E8.5, these regions broadly express other transcription factors, the future midbrain expresses *Wnt1* together with *Otx2*, and the future anterior hindbrain (R1 of metencephalon) expresses *Fgf8* and *Gbx2.* The transcription factors engrailed, *En1* and *En2*, and the paired box, *Pax2* and *Pax5*, at this stage are expressed in the entire mesencephalon and rhombomere 1.[106] Later at E9.5, some transcription factors become restricted to the *Otx2/Gbx2* border: *Wnt1* to the narrow anterior section and the *Pax2*, *Fgf8* and *Gbx2* to a posterior section. *Wnt1* maintains its expression also in the dorsal mesencephalon. *En1*, *En2* and *Pax5* also continue their expression.[106,116] At this stage, the localisation of the organisers becomes restricted to specific locations: both FGF8 and WNT1 in the *Otx2/Gbx2* border, and WNT1 in the dorsal mesencephalon (reviewed elsewhere[114,115,118,119])

Hox-mediated hindbrain patterning and retinoic acid induction Hox genes confer anterior-posterior positional information to the neuronal precursors located caudally to the *Otx2/Gbx2* border, in the prospective hindbrain region. It has been postulated that this information, at each rhombomere, can be represented as a Hox code of specific combinatorial expression.[120–122] *Hox* genes are found in all animal species with conserved roles in body patterning.[123] Most of the knowledge of their function in vertebrates derives from knockout studies in mice or manipulation of their

activity in chick embryos.[105]

In most vertebrates, there are 39 *Hox* genes which contain a region that encodes for a homeodomain that mediates DNA binding. These genes are mostly expressed in the CNS and are distributed across 4 clusters: the *HoxA, HoxB, HoxC* and *HoxD*, containing 13 paralog groups each (*Hox1-Hox13*).[105] *Hox1-Hox5* paralog group genes are expressed in the hindbrain and some of them over a narrow time window during development, while others may persist to postnatal stages.[105]

In mice, *Hox* gene expression in the brainstem begins as early as E7.5, with the expression of the Hox1 paralogs, *Hoxa1* and *Hoxb1*, followed by the paralogs *Hox2* and *Hox3* at E8.5.[105,124] Mice knockout studies suggest that early expression of *Hox1* and *Hox2* genes are determinant for the correct compartmentalisation of the hindbrain.[105,125,126]

Although different neuronal populations are generated within the hindbrain, the study of the lineages of hindbrain motor neurons provides a valuable way to study the function of Hox genes. Motor neurons are generated in specific ventral locations throughout the hindbrain rhombomeres and generally cluster in motor nuclei that project to various body locations as cranial nerves. Similar to motor neurons, the hindbrain non-motor neurons require a complex interplay between multiple *Hox* genes, which are crucial to determine their correct identity and connectivity[105,127–129] (reviewed elsewhere[105]).

In the hindbrain, *Hox* genes are active in neuronal progenitors and it appears that they can also impinge on dorsal-ventral fate specification programs.[105,130] No such role has yet been described in the spinal cord and this difference may be related with to distinct temporal and spatial profiles of Hox genes in each region, which provides an additional cue in the diversification of the neuronal populations in the hindbrain.[105]

Retinoic acid patterning starts early in mice and helps to establish the anterior-posterior axis of the hindbrain. Its importance has been supported by the existence of retinoid synthetic and analytic enzymes, binding proteins and receptors in the hindbrain. Also, it has been shown that Hox genes and other developmental regulatory genes, are profoundly influenced by retinoid acid signalling.[131–137]

In mouse, it has been shown that retinoic acid is produced and secreted by the paraxial mesoderm corresponding to the first four to six somites. These centres lie adjacent to the most posterior rhombomeres (R7-8), which indicates a higher concentration in this posterior location. Thus, retinoic acid signals according to a posterior-to-anterior gradient (reviewed elsewhere[137]). The distribution of the principal retinoic acid synthetic enzyme, retinaldehyde dehydrogenase 2, within the most anterior somites (adjacent to posterior rhombomeres) and the existence of retinoic acid degrading enzymes, like cytochrome P450 of the family CYP26, in the anterior rhombomeres (R2-5), help explain the generation of a signalling gradient.[137–142]

An appropriate patterning of the hindbrain and generation of an axial expression of Hox genes, requires a correct spatial gradient and timed availability of retinoic acid. Nevertheless, it is important to consider the simultaneous inductions of retinoic acid and morphogenes from the FGF family, the FGF4 from the somites at caudal hindbrain and FGF8 from the isthmus at rostral hindbrain.[137] An *opposing signal* model tries to correlate a series of mutual interactive loops between FGF morphogene induction, retinoic acid receptors, and *Hox* genes in order to unravel the regulatory mechanisms behind Hox gene expression.[137,143]

3.2.2 Anterior-posterior segmentation of vulnerable populations in Parkinson's disease (prosomeric model - Supplementary figure 1 and table 1)

The correct localization of the isthmus organizer in the mid-hindbrain border and the hox-mediated anterior-posterior patterning of the hindbrain are among the earliest events responsible for specification of brainstem's neuronal progenitors. Therefore, the correct anterior-posterior specification have already been studied for multiple brainstem populations (Supplementary table 1) (e.g. 10N,[10,137] raphe nuclei,[19,144] LC[43,44,144–152] and SNC[68,106,116,117,144,153–177]).

<table>
<tr><th></th><th>P2/1</th><th>M1/2</th><th>R1</th><th>R2</th><th>R3</th><th>R4</th><th>R5</th><th>R6</th><th>R7</th><th>R8</th></tr>
<tr><td>Otx2[154, 156, 157, 159, 160, 178]</td><td colspan="2">+</td><td colspan="8">-</td></tr>
<tr><td>Gbx2[106, 116, 117, 168, 171, 172, 175]</td><td colspan="2">-</td><td colspan="3">+</td><td colspan="5">-</td></tr>
<tr><td>Emx2[179]</td><td colspan="2">+</td><td colspan="8"></td></tr>
<tr><td>En1/2[106, 162–167]</td><td>-</td><td>+</td><td>+</td><td colspan="7">-</td></tr>
<tr><td>Egr2[10, 124, 127, 180]</td><td colspan="4">-</td><td>+</td><td>-</td><td>+</td><td colspan="3">-</td></tr>
<tr><td>Mafb[10, 124, 127, 180]</td><td colspan="6">-</td><td colspan="4">+</td></tr>
<tr><td>Nr2f1[127]</td><td colspan="7">-</td><td colspan="3">+</td></tr>
<tr><td>Hoxa1[10, 105, 127]</td><td colspan="5">-</td><td colspan="5">+</td></tr>
<tr><td>Hoxb1[10, 105, 115, 127]</td><td colspan="5">-</td><td colspan="5">+</td></tr>
<tr><td>Hoxa2[10, 105, 124, 127, 180]</td><td colspan="3">-</td><td colspan="7">+</td></tr>
<tr><td>Hoxb2[10, 105, 124, 127, 180]</td><td colspan="4">-</td><td colspan="6">+</td></tr>
<tr><td>Hoxa3[10, 105, 124, 127]</td><td colspan="6">-</td><td colspan="4">+</td></tr>
<tr><td>Hoxb3[10, 105, 124, 127, 180]</td><td colspan="6">-</td><td colspan="4">+</td></tr>
<tr><td>Hoxd3[10, 105, 124, 127, 154]</td><td colspan="6">-</td><td colspan="4">+</td></tr>
<tr><td>Hoxa4[10, 105, 124, 127]</td><td colspan="8">-</td><td colspan="2">+</td></tr>
<tr><td>Hoxb4[10, 105, 124, 127, 180]</td><td colspan="8">-</td><td colspan="2">+</td></tr>
<tr><td>Hoxd4[10, 105, 124, 127]</td><td colspan="8">-</td><td colspan="2">+</td></tr>
<tr><td>Hoxc5[105]</td><td colspan="8">-</td><td>-</td><td>+</td></tr>
</table>

Supplementary table 1: Brainstem segmentation program. Required developmental transcription factors during the anterior-posterior segmentation program of the brainstem. (+) required to be expressed, (-) required not to be expressed, (empty) out of knowledge, no data available.

3.2.3 Dorsal-ventral patterning and neuronal induction:

Dorsal-ventral patterning mainly results from the integration of three instructive cues from SHH,[181] WNTs and BMPs.[99] These signalling molecules are produced and secreted by two opposing organisational centres, which locate in the most ventral and dorsal positions of the neural tube. SHH is produced and secreted by the floor plate and notochord and instructs ventral identities. In turn, the WNTs and BMPs are produced in the roof plate and signal opposing dorsal identities.[99]

Before closure of the neural tube, the neural plate already has a dorsal identity, characterised by the expression of *Pax3* and *Pax7*,[99] which however is not sufficient for neural progenitors to differentiate as dorsal neural sub-types.[99] Most of the studies that seek to understand the mechanisms behind the progressive acquisition of neural cell identity in the dorsal-ventral patterning have been made in mouse and chick spinal cord, since it is the simplest and most conserved structure in vertebrate CNS.[99] As a result of these patterning events in the developing spinal cord, it's possible to distinguish 11 discrete domains of neuronal progenitors, with 5 ventral (p3, pMN, p2-0 from ventral to dorsal) and six dorsal domains (dP1-6 from dorsal to ventral). These domains can be identified by a particular combination of transcription factors, which will determine the type of neuronal progenitor to be produced.[99, 100]

Sonic hedgehog signalling SHH is a morphogen secreted by the ventral floor plate[171] and notochord (underlying the floor plate) and is both necessary and sufficient to induce a ventral neural fate.[99] Its secretion begins around E8.5 in mouse and the ventral-dorsal gradient patterns ventral locations in a concentration-dependent way.[99]

Along with neural tube closure, the floor plate SHH signalling promotes the expression of the ventral determinants, which lead to in the repression of *Pax3/7* that become restricted to dorsal populations.[99] The molecular mechanism behind SHH signalling and the consequent induction of intracellular cell fate decisions have been addressed in many studies that focus on signal recognition, interpretation and transformation.[99, 100, 103, 182–186] The analysis of mouse and chick spinal cords,

where *Shh* is expressed by the floor plate, lead to a comprehensive understanding of its signalling mechanism.[100]

All regions of the nervous system that receive ventral induction by SHH have the same activator and repressor combinations, to a different extent, which generate the appropriate cellular responses.[100] Positive SHH signalling induces the expression of *Gli2* and (at a lesser extent) *Gli3* activators, which induce the expression of the Gli1 activator,[187] and in its absence, the *Gli3* repressor is expressed (reviewed elsewhere[100]). Through the *Gli* family, ventral induction results in the expression/repression of various downstream transcription factors within progenitors. In this signalling cascade, the expression of class I homeobox transcription factors *Pax6*, *Pax7*, *Irx3* and *Dbx1/2* is repressed and the class II homeobox transcription factors *Nkx2-2*[188] and *Nkx6-1* are selectively expressed.

Cross-repressive interactions between downstream transcription factors are responsible for directing cell differentiation into specific cell sub-types.[100] The mechanistic link between GLI proteins and the resultant patterns of homeodomain transcription factor expression remains to be elucidated, although the role of SHH signalling in the ventral patterning has been studied for different regions of the developing vertebrate CNS.[100] SHH controls the sorting of the distinct progenitor groups through *Gli* transcription mediators.[100] Furthermore, SHH also has a developmental role in the generation of oligodendrocytes and a mitogenic role in controlling the proliferation of neural progenitors. At later stages, SHH acts in axonal pathfinding and maintenance of adult stem cells.[100] The specific actions of these diverse functions are however dependent on space and time[100].

WNT and BMP signalling WNTs regulate cell-to-cell interactions during embryogenesis by WNT-frizzled signal transduction.[99,189] During neurulation, in a narrow temporal window from 9.5 to 11.5 days post conception,[190] WNT is also produced and secreted dorsally in the roof plate[99] and in the isthums organiser. Its signalling antagonises the ventral SHH signalling (SHH/GLI)[99] and is currently understood to be dependent on receptors of the Frizzed family located, on the cell surface. The molecular mechanism of its signal transduction involves multiple cytoplasmic components, in the canonical pathway, that stabilises beta-catenin, which enters the nucleus and forms a complex with T-cell Factors (TCFs) that further activate the expression of specific transcription factors.[99,189] In mouse, several WNTs, including WNT1 and WNT3A, are produced in the roof plate and surrounding areas and although they have been primarily considered as mitogenic signals, recent studies highlighted their importance in neural tube patterning.[99] LRP6, frizzled class receptors (such as FDZ3), WNT2, DKK1, are also important for WNT signalling and their knock-out leads to a delay in differentiation or neuronal loss.[68]

BMPs are a subgroup of the TGF-beta super-family of secreted proteins that signal through complexes of transmembrane serine/threonine kinases.[99] Canonical BMP signalling leads to the phosphorylation of the intracellular factors SMAD1/5/8 that further interact with SMAD4 to form a stabilised complex. This complex, together with additional cofactors, regulate the transcription of specific target genes.[99] Before neural tube closure, BMP2 and BMP4 are expressed in the epidermal ectoderm and BMP4 and BMP5 are expressed in the neural fold. At early stages, BMP7 is expressed in the epidermal ectoderm and in the notochord. After neural tube closure, BMP4, BMP5 and BMP7, as well as other proteins of the TGF-beta super-family, such as GDF7, DSL-1 and Activin a/b, are expressed in the roof plate. During primary neurogenesis of dorsal inter-neurons (dINs), the expression of BMP7 extends throughout the dorsal half of the neural tube[99] (dependence on BMP4 and BMP7 reviewed elsewhere[99]).

Its has been suggested that BMP activity lies upstream of the WNT pathway.[99] In a model that consider triple induction, the main contribution of BMP signalling is to stimulate the expression of WNTs, especially ligands, which initiate the activity of the WNT pathway, which in turn modulate the expression of the main transcriptional effectors of the SHH pathway.[99] The interplay between WNT and SHH signalling plays an important role in defining the neuronal identity of populations, such as midbrain DN.[190]

3.2.4 Dorsal-ventral patterning and specification program of vulnerable populations in Parkinson's disease (neuronal specification - Supplementary figure 1 and table 2)

Transcription factors	P2-P1 SNC(A9) /VTA(A10)	M1-M2 SNC(A9) /VTA(A10)	R1-4 LC(A6)/SubC(A4)	R1 DR(B6-7) /PnR(B5)	R2-3 MnR(B8)/ PnR(B5) R5-8 RM(B3)/ RP(B2)/ RO(B1)	R7-8 10N
Gli1/2/3	(+)[191–193]	(+)[187,191–196]	(-)[187,197]	(+)[192,198]		(+)[10,15,183,198–200]
Foxa1	(+)[201]	(+)[201,202]	(-)[187,197]			
Foxa2	(+)[201]	(+)[68,195,201–204]	(-)[187,197]	(+)[36,205]	(+)[205,206]	(-)[205]
Ascl1	(+)*[202]		(-)[44,207]	(+)[208]		(+)*[209]
Lmx1a	(+)[201,210,211]	(+)[159,195,201,210–213]				
Lmx1b	(+)[201]	(+)[159,195,201,212–216]	(+)[36,217,218]	(+)[36,217,218]		
Gata2			(-)[219]	(+)[19,206,220]		
Gata3			(-)[219]	(+)[19,206,220,221]	(+)[19,206,221]	
Phox2a			(+)[44,207,222,223]			(+)*[10,224,225]
Phox2b			(+)[42,44,46,226]		(-)[206]	(+)[10,224,225]
Tlx3			(+)[46,227]			
Msx1	(+)*[211]	(+)[211,228]				
Nkx2-2	(-)[201]		(-)[187,197]	(+)[36,188,220]	(+)[188,206]	(+)[9,10,182,184,188,229,230]
Nkx2-9			(-)[187,197]		(-)[206]	(+)[9,10,182,184,188,229]
Nkx6-1	(-)[211]		(-)[187,197]	(+)[36,220,231]		(+)*[10,184,232,233]
Nkx6-2			(-)[187,197]			(+)*[10,184,232,233]
Neurog2	(+)[202]	(+)[159,160,202,211,234–236]				
Onecut1/2/3	(+)[237]		(+)[46]			
Isl1						(+)[10,238–241]
Isl2						(-)[10,238–241]
Insm1			(+)[46,242]	(+)[242]		
Nr2f6			(+)[243]			
Pet1				(+)[19,28,217,244]		
Nr4a2	(+)[210,211,245–248]	(+)[159,210,211,245–252]				
Pitx3	(+)[57,158,237,246–248,253,254]	(+)[57,158,172,237,246–248,253–258]				
Tbx20						(+)[10,259]
Lhx3/4						(-)[10,238]
Dbx1/2						(-)[231,232]
Irx3						(-)[260,261]

Supplementary table 2: Neuronal specification of some brainstem populations vulnerable in PD. Required developmental transcription factors (or involved*) during the neuronal specification program of some vulnerable brainstem neuronal populations in PD. (+) required to be expressed, (-) required not to be expressed, (empty) out of knowledge, no data available.

3.2.5 Similarities between developmental requirements

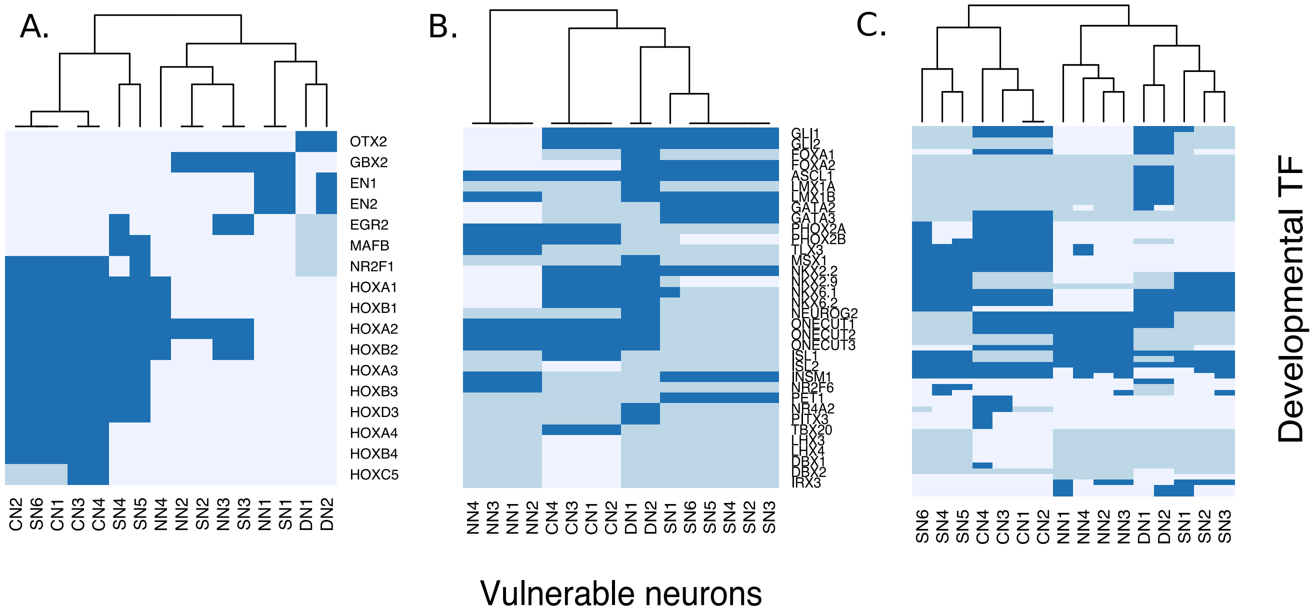

Supplementary figure 1: Clustered matrix of developmental transcription factors required to be active or inactive in the lineage specification of some selectively vulnerable neurons in Parkinson's disease. A. Anterior-posterior segmentation. B. Dorsal-ventral patterning and specification program. C. Both A and B requirements. Dark blue (required expression), white (required no expression) and light blue (no information available). CN [cholinergic visceromotor neurons of the dorsal motor nucleus of the vagus (10N with cR8-10 origin)], DN [dopaminergic neurons of the substantia nigra pars compacta (A9 group with M1-2 and P2-1 origin) and ventral tegmental area (A10 group with M1-2 origin)], NN [noradrenergic neurons of the locus coeruleus (A6 group with R1 origin)], SN [serotonergic neurons of the upper/pontine raphe nuclei (B5-8 groups with R1-3 origin), and lower/medullary raphe nuclei (B1-3 groups with R5-7 origin).

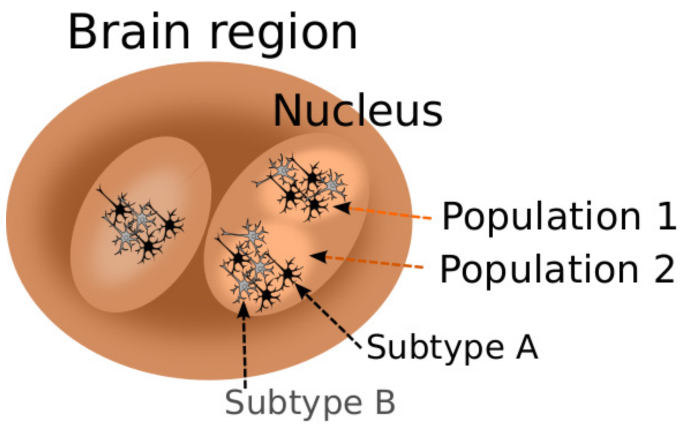

Supplementary figure 2: Definition of brain region, nucleus, neuronal populations and neuronal subtypes

References

[1] Sulzer, D. and Surmeier, D. J. Neuronal vulnerability, pathogenesis, and Parkinson's disease: Neuronal Vulnerability, Pathogenesis, and PD. *Movement Disorders* **28**(6), 715–724, June (2013).

[2] Del Tredici, K., Rüb, U., De Vos, R. A. I., Bohl, J. R. E., and Braak, H. Where does parkinson disease pathology begin in the brain? *Journal of neuropathology and experimental neurology* **61**(5), 413–426, May (2002).

[3] Braak, H., Tredici, K. D., Rüb, U., de Vos, R. A., Jansen Steur, E. N., et al. Staging of brain pathology related to sporadic Parkinson's disease. *Neurobiology of Aging* **24**(2), 197–211, March (2003).

[4] Braak, H. and Tredici, K. D. *Neuroanatomy and Pathology of Sporadic Parkinson's Disease.* Springer, , November (2008).

[5] Cheng, G., Zhu, H., Zhou, X., Qu, J., Ashwell, K. W. S., et al. Development of the human dorsal nucleus of the vagus. *Early Human Development* **84**(1), 15–27, January (2008).

[6] Huang, X. F., Törk, I., and Paxinos, G. Dorsal motor nucleus of the vagus nerve: a cyto- and chemoarchitectonic study in the human. *The Journal of comparative neurology* **330**(2), 158–182, April (1993).

[7] Phelps, P. E., Brennan, L. A., and Vaughn, J. E. Generation patterns of immunocytochemically identified cholinergic neurons in rat brainstem. *Developmental Brain Research* **56**(1), 63–74, October (1990).

[8] Rinaman, L. and Levitt, P. Establishment of vagal sensorimotor circuits during fetal development in rats. *Journal of Neurobiology* **24**(5), 641–659, May (1993).

[9] Jacob, J., Hacker, A., and Guthrie, S. Mechanisms and molecules in motor neuron specification and axon pathfinding. *BioEssays* **23**(7), 582–595 (2001).

[10] Guthrie, S. Patterning and axon guidance of cranial motor neurons. *Nature Reviews Neuroscience* **8**(11), 859–871, November (2007).

[11] Weihe, E., Depboylu, C., Schütz, B., Schäfer, M. K.-H., and Eiden, L. E. Three Types of Tyrosine Hydroxylase-Positive CNS Neurons Distinguished by Dopa Decarboxylase and VMAT2 Co-Expression. *Cellular and molecular neurobiology* **26**(0), 659–678 (2006).

[12] Koutcherov, Y., Halliday, G., Paxinos, G., and Huang, X.-F. Chapter 10 - Organization of Human Brain Stem Nuclei. In The Human Nervous System (Second Edition), Paxinos, G. and Mai, J. K., editors, 267–320. Academic Press, San Diego (2004).

[13] Chang, H. Y., Mashimo, H., and Goyal, R. K. IV. Current concepts of vagal efferent projections to the gut. *American Journal of Physiology - Gastrointestinal and Liver Physiology* **284**(3), G357–G366, March (2003).

[14] Ratcliffe, E. M., Farrar, N. R., and Fox, E. A. Development of the Vagal Innervation of the GUT: Steering the Wandering Nerve. *Neurogastroenterology and motility : the official journal of the European Gastrointestinal Motility Society* **23**(10), 898–911, October (2011).

[15] Chandrasekhar, A. Turning Heads: Development of Vertebrate Branchiomotor Neurons. *Developmental dynamics : an official publication of the American Association of Anatomists* **229**(1), 143–161, January (2004).

[16] Halliday, G. M., Li, Y. W., Blumbergs, P. C., Joh, T. H., Cotton, R. G., et al. Neuropathology of immunohistochemically identified brainstem neurons in Parkinson's disease. *Annals of Neurology* **27**(4), 373–385, April (1990).

[17] Gai, W. P., Halliday, G. M., Blumbergs, P. C., Geffen, L. B., and Blessing, W. W. Substance P-Containing Neurons in the Mesopontine Tegmentum Are Severely Affected in Parkinson's Disease. *Brain* **114**(5), 2253–2267, October (1991).

[18] Wylie, C. J., Hendricks, T. J., Zhang, B., Wang, L., Lu, P., et al. Distinct Transcriptomes Define Rostral and Caudal Serotonin Neurons. *The Journal of Neuroscience* **30**(2), 670–684, January (2010).

[19] Alonso, A., Merchan, P., Sandoval, J. E., Sanchez-Arrones, L., Garcia-Cazorla, A., et al. Development of the serotonergic cells in murine raphe nuclei and their relations with rhombomeric domains. *Brain Structure & Function* **218**, 1229–1277 (2013).

[20] Mason, P. Contributions of the Medullary Raphe and Ventromedial Reticular Region to Pain Modulation and Other Homeostatic Functions. *Annual Review of Neuroscience* **24**(1), 737–777 (2001).

[21] Erickson, J. T., Shafer, G., Rossetti, M. D., Wilson, C. G., and Deneris, E. S. Arrest of 5ht neuron differentiation delays respiratory maturation and impairs neonatal homeostatic responses to environmental challenges. *Respiratory Physiology & Neurobiology* **159**(1), 85–101, October (2007).

[22] Zhao, Z.-Q., Chiechio, S., Sun, Y.-G., Zhang, K.-H., Zhao, C.-S., et al. Mice Lacking Central Serotonergic Neurons Show Enhanced Inflammatory Pain and an Impaired Analgesic Response to Antidepressant Drugs. *The Journal of Neuroscience* **27**(22), 6045–6053, May (2007).

[23] Gargaglioni, L. H., Bícego, K. C., and Branco, L. G. S. Brain monoaminergic neurons and ventilatory control in vertebrates. *Respiratory Physiology & Neurobiology* **164**(1-2), 112–122, December (2008).

[24] Hodges, M. R., Tattersall, G. J., Harris, M. B., McEvoy, S. D., Richerson, D. N., et al. Defects in Breathing and Thermoregulation in Mice with Near-Complete Absence of Central Serotonin Neurons. *The Journal of Neuroscience* **28**(10), 2495–2505, March (2008).

[25] Lucki, I. The spectrum of behaviors influenced by serotonin. *Biological psychiatry* **44**(3), 151–162 (1998).

[26] Sodhi, M. S. and Sanders-Bush, E. Serotonin and brain development. *International review of neurobiology* **59**, 111–174 (2004).

[27] Jacobs, B. L. and Azmitia, E. C. Structure and function of the brain serotonin system. *Physiological Reviews* **72**(1), 165–229, January (1992).

[28] Hendricks, T. J., Fyodorov, D. V., Wegman, L. J., Lelutiu, N. B., Pehek, E. A., et al. Pet-1 ETS Gene Plays a Critical Role in 5-HT Neuron Development and Is Required for Normal Anxiety-like and Aggressive Behavior. *Neuron* **37**(2), 233–247, January (2003).

[29] Lerch-Haner, J. K., Frierson, D., Crawford, L. K., Beck, S. G., and Deneris, E. S. Serotonergic transcriptional programming determines maternal behavior and offspring survival. *Nature neuroscience* **11**(9), 1001–1003, September (2008).

[30] Ansorge, M. S., Hen, R., and Gingrich, J. A. Neurodevelopmental origins of depressive disorders. *Current Opinion in Pharmacology* **7**(1), 8–17, February (2007).

[31] Lesch, K.-P., Araragi, N., Waider, J., Hove, D. v. d., and Gutknecht, L. Targeting brain serotonin synthesis: insights into neurodevelopmental disorders with long-term outcomes related to negative emotionality, aggression and antisocial behaviour. *Philosophical Transactions of the Royal Society of London B: Biological Sciences* **367**(1601), 2426–2443, September (2012).

[32] Haavik, J., Blau, N., and Thöny, B. Mutations in human monoamine-related neurotransmitter pathway genes. *Human Mutation* **29**(7), 891–902, July (2008).

[33] Holmes, A. Genetic variation in cortico-amygdala serotonin function and risk for stress-related disease. *Neuroscience & Biobehavioral Reviews* **32**(7), 1293–1314, September (2008).

[34] Waider, J., Araragi, N., Gutknecht, L., and Lesch, K.-P. Tryptophan hydroxylase-2 (TPH2) in disorders of cognitive control and emotion regulation: A perspective. *Psychoneuroendocrinology* **36**(3), 393–405, April (2011).

[35] Homberg, J. R. and Lesch, K.-P. Looking on the Bright Side of Serotonin Transporter Gene Variation. *Biological Psychiatry* **69**(6), 513–519, March (2011).

[36] Kiyasova, V. and Gaspar, P. Development of raphe serotonin neurons from specification to guidance. *European Journal of Neuroscience* **34**(10), 1553–1562, November (2011).

[37] Zweig, R. M., Cardillo, J. E., Cohen, M., Giere, S., and Hedreen, J. C. The locus ceruleus and dementia in Parkinson's disease. *Neurology* **43**(5), 986–991, May (1993).

[38] Greenfield, J. G. and Bosanquet, F. D. The Brain-Stem Lesions in Parkinsonism. *Journal of Neurology, Neurosurgery & Psychiatry* **16**(4), 213–226, November (1953).

[39] Halliday, G. Chapter 14 - Substantia Nigra and Locus Coeruleus. In The Human Nervous System (Second Edition), Paxinos, G. and Mai, J. K., editors, 449–463. Academic Press, San Diego (2004).

[40] Pierce, E. T. Time of Origin of Neurons in the Brain Stem of the Mouse. In Progress in Brain Research, Ford, D. H., editor, volume 40 of *Neurobiological Aspects of Maturation and Aging*, 53–65. Elsevier (1973).

[41] Steindler, D. A. and Trosko, B. K. Two types of locus coeruleus neurons born on different embryonic days in the mouse. *Anatomy and embryology* **179**(5), 423–434 (1989).

[42] Pattyn, A., Goridis, C., and Brunet, J.-F. Specification of the Central Noradrenergic Phenotype by the Homeobox Gene Phox2b. *Molecular and Cellular Neuroscience* **15**(3), 235–243, March (2000).

[43] Vogel-Höpker, A. and Rohrer, H. The specification of noradrenergic locus coeruleus (LC) neurones depends on bone morphogenetic proteins (BMPs). *Development* **129**(4), 983–991, February (2002).

[44] Goridis, C. and Rohrer, H. Specification of catecholaminergic and serotonergic neurons. *Nature Reviews Neuroscience* **3**(7), 531–541, July (2002).

[45] Aroca, P., Lorente-Cánovas, B., Mateos, F. R., and Puelles, L. Locus coeruleus neurons originate in alar rhombomere 1 and migrate into the basal plate: Studies in chick and mouse embryos. *The Journal of Comparative Neurology* **496**(6), 802–818, June (2006).

[46] Espana, A. and Clotman, F. Onecut factors control development of the Locus Coeruleus and of the mesencephalic trigeminal nucleus. *Molecular and Cellular Neuroscience* **50**(1), 93–102, May (2012).

[47] Shi, M., Guo, C., Dai, J.-X., and Ding, Y.-Q. DCC is required for the tangential migration of noradrenergic neurons in locus coeruleus of mouse brain. *Molecular and Cellular Neuroscience* **39**(4), 529–538, November (2008).

[48] Allen Institute for Brain Science. Allen Developing Mouse Brain Atlas, (2013). 00000.

[49] Saper, C. B. 'Like a thief in the night' : the selectivity of degeneration in Parkinson's disease. *Brain* **122**(8), 1401–1402, August (1999).

[50] Rubenstein, J. and Rakic, P. *Patterning and Cell Type Specification in the Developing CNS and PNS: Comprehensive Developmental Neuroscience*. Academic Press, , May (2013).

[51] Zarow C, Lyness SA, Mortimer JA, and Chui HC. Neuronal loss is greater in the locus coeruleus than nucleus basalis and substantia nigra in alzheimer and parkinson diseases. *Archives of Neurology* **60**(3), 337–341, March (2003).

[52] Hilker R, Schweitzer K, Coburger S, and et al. NOnlinear progression of parkinson disease as determined by serial positron emission tomographic imaging of striatal fluorodopa f 18 activity. *Archives of Neurology* **62**(3), 378–382, March (2005).

[53] Fahn, S. and Sulzer, D. Neurodegeneration and Neuroprotection in Parkinson Disease. *NeuroRx* **1**(1), 139–154, January (2004).

[54] Damier, P., Hirsch, E. C., Agid, Y., and Graybiel, A. M. The substantia nigra of the human brain II. Patterns of loss of dopamine-containing neurons in Parkinson's disease. *Brain* **122**(8), 1437–1448, August (1999).

[55] Damier, P., Hirsch, E. C., Agid, Y., and Graybiel, A. M. The substantia nigra of the human brain. I. Nigrosomes and the nigral matrix, a compartmental organization based on calbindin D(28k) immunohistochemistry. *Brain* **122**(8), 1421–1436, August (1999).

[56] Double, K., Reyes, S., Werry, E., and Halliday, G. Selective cell death in neurodegeneration: Why are some neurons spared in vulnerable regions? *Progress in Neurobiology* **92**(3), 316–329, November (2010).

[57] Alavian, K. N., Scholz, C., and Simon, H. H. Transcriptional regulation of mesencephalic dopaminergic neurons: The full circle of life and death. *Movement Disorders* **23**(3), 319–328, February (2008).

[58] Betarbet, R., Sherer, T. B., Mackenzie, G., Garcia-osuna, M., Panov, A. V., et al. Chronic systemic pesticide exposure reproduces features of Parkinson's disease. *Nature Neuroscience* **3**(12), 1301–1306 (2000).

[59] Farrer, M. J. Genetics of Parkinson disease: paradigm shifts and future prospects. *Nature Reviews Genetics* **7**(4), 306–318, April (2006).

[60] McNaught, K. S. P., Perl, D. P., Brownell, A.-L., and Olanow, C. W. Systemic exposure to proteasome inhibitors causes a progressive model of Parkinson's disease. *Annals of Neurology* **56**(1), 149–162, July (2004).

[61] Hirsch, E., Graybiel, A. M., and Agid, Y. A. Melanized dopaminergic neurons are differentially susceptible to degeneration in Parkinson's disease. *Nature* **334**(6180), 345–348, July (1988).

[62] Grimm, J., Mueller, A., Hefti, F., and Rosenthal, A. Molecular basis for catecholaminergic neuron diversity. *Proceedings of the National Academy of Sciences of the United States of America* **101**(38), 13891–13896, September (2004).

[63] Brown, M. T. C., Henny, P., Bolam, J. P., and Magill, P. J. Activity of Neurochemically Heterogeneous Dopaminergic Neurons in the Substantia Nigra during Spontaneous and Driven Changes in Brain State. *Journal of Neuroscience* **29**(9), 2915–2925, March (2009).

[64] Smits, S. M., von Oerthel, L., Hoekstra, E. J., Burbach, J. P. H., and Smidt, M. P. Molecular marker differences relate to developmental position and subsets of mesodiencephalic dopaminergic neurons. *PloS one* **8**(10), e76037 (2013).

[65] Björklund, A. and Dunnett, S. B. Dopamine neuron systems in the brain: an update. *Trends in Neurosciences* **30**(5), 194–202, May (2007).

[66] Korotkova, T. M., Ponomarenko, A. A., Brown, R. E., and Haas, H. L. Functional diversity of ventral midbrain dopamine and GABAergic neurons. *Molecular Neurobiology* **29**(3), 243–259, June (2004).

[67] Greene, J. G., Dingledine, R., and Greenamyre, J. T. Gene expression profiling of rat midbrain dopamine neurons: implications for selective vulnerability in parkinsonism. *Neurobiology of Disease* **18**(1), 19–31, February (2005).

[68] Hegarty, S. V., Sullivan, A. M., and O'Keeffe, G. W. Midbrain dopaminergic neurons: A review of the molecular circuitry that regulates their development. *Developmental Biology* **379**(2), 123–138, July (2013).

[69] Saper, C. B., Sorrentino, D. M., German, D. C., and de Lacalle, S. Medullary catecholaminergic neurons in the normal human brain and in Parkinson's disease. *Annals of Neurology* **29**(6), 577–584, June (1991).

[70] Gibb, W. R. G. Melanin, tyrosine hydroxylase, calbindin and substance P in the human midbrain and substantia nigra in relation to nigrostriatal projections and differential neuronal susceptibility in Parkinson's disease. *Brain Research* **581**(2), 283–291, May (1992).

[71] Dopeso-Reyes, I. G., Rico, A. J., Roda, E., Sierra, S., Pignataro, D., et al. Calbindin content and differential vulnerability of midbrain efferent dopaminergic neurons in macaques. *Frontiers in Neuroanatomy* **8**, December (2014).

[72] Liang, C. L., Sinton, C. M., Sonsalla, P. K., and German, D. C. Midbrain dopaminergic neurons in the mouse that contain calbindin-D28k exhibit reduced vulnerability to MPTP-induced neurodegeneration. *Neurodegeneration: a journal for neurodegenerative disorders, neuroprotection, and neuroregeneration* **5**(4), 313–318, December (1996).

[73] Liang, C.-L., Nelson, O., Yazdani, U., Pasbakhsh, P., and German, D. C. Inverse relationship between the contents of neuromelanin pigment and the vesicular monoamine transporter-2: Human midbrain dopamine neurons. *The Journal of Comparative Neurology* **473**(1), 97–106, May (2004).

[74] van Domburg, P. H. and ten Donkelaar, H. J. The human substantia nigra and ventral tegmental area. A neuroanatomical study with notes on aging and aging diseases. *Advances in Anatomy, Embryology, and Cell Biology* **121**, 1–132 (1991).

[75] Braak, H. and Braak, E. Nuclear configuration and neuronal types of the nucleus niger in the brain of the human adult. *Human Neurobiology* **5**(2), 71–82 (1986).

[76] Di Porzio, U., Zuddas, A., Cosenza-Murphy, D. B., and Barker, J. L. Early appearance of tyrosine hydroxylase immunoreactive cells in the mesencephalon of mouse embryos. *International Journal of Developmental Neuroscience: The Official Journal of the International Society for Developmental Neuroscience* **8**(5), 523–532 (1990).

[77] Toulouse, A. and Sullivan, A. M. Progress in Parkinson's disease—Where do we stand? *Progress in Neurobiology* **85**(4), 376–392, August (2008).

[78] McRitchie, D. A., Hardman, C. D., and Halliday, G. M. Cytoarchitectural distribution of calcium binding proteins in midbrain dopaminergic regions of rats and humans. *The Journal of Comparative Neurology* **364**(1), 121–150, January (1996).

[79] Wichterle, H., Gifford, D., and Mazzoni, E. Mapping Neuronal Diversity One Cell at a Time. *Science* **341**(6147), 726–727, August (2013).

[80] Tau, G. Z. and Peterson, B. S. Normal Development of Brain Circuits. *Neuropsychopharmacology* **35**(1), 147–168, January (2010).

[81] Flames, N. and Hobert, O. Transcriptional Control of the Terminal Fate of Monoaminergic Neurons. *Annual Review of Neuroscience* **34**(1), 153–184 (2011).

[82] Copp, A. J., Greene, N. D. E., and Murdoch, J. N. The genetic basis of mammalian neurulation. *Nature reviews. Genetics* **4**(10), 784–793, October (2003).

[83] Müller, F. and O'Rahilly, R. Chapter 2 - Embryonic Development of the Central Nervous System. In The Human Nervous System (Second Edition), Paxinos, G. and Mai, J. K., editors, 22–48. Academic Press, San Diego (2004).

[84] Copp, A. J. Neurulation in the cranial region - normal and abnormal. *Journal of Anatomy* **207**(5), 623–635, November (2005).

[85] Shum, A. S. and Copp, A. J. Regional differences in morphogenesis of the neuroepithelium suggest multiple mechanisms of spinal neurulation in the mouse. *Anatomy and Embryology* **194**(1), 65–73, July (1996).

[86] Morriss-Kay, G. M. Growth and development of pattern in the cranial neural epithelium of rat embryos during neurulation. *Journal of Embryology and Experimental Morphology* **65 Suppl**, 225–241, October (1981).

[87] Lawrence, N. and Morel, V. Dorsal closure and convergent extension: two polarised morphogenetic movements controlled by similar mechanisms? *Mechanisms of Development* **120**(11), 1385–1393, November (2003).

[88] Altmann, C. R. and Brivanlou, A. H. Neural patterning in the vertebrate embryo. In International Review of Cytology, Lawrence D. Etkin, K. W. J., editor, volume Volume 203, 447–482. Academic Press (2001).

[89] McKay, R. Stem cells in the central nervous system. *Science (New York, N.Y.)* **276**(5309), 66–71, April (1997).

[90] Rao, M. S. Multipotent and restricted precursors in the central nervous system. *The Anatomical Record* **257**(4), 137–148, August (1999).

[91] Leone, D. P., Relvas, J. B., Campos, L. S., Hemmi, S., Brakebusch, C., et al. Regulation of neural progenitor proliferation and survival by $\beta 1$ integrins. *Journal of Cell Science* **118**(12), 2589–2599, June (2005).

[92] Anderson, D. J. Stem cells and pattern formation in the nervous system: the possible versus the actual. *Neuron* **30**(1), 19–35, April (2001).

[93] Gaiano, N. and Fishell, G. The Role of Notch in Promoting Glial and Neural Stem Cell Fates. *Annual Review of Neuroscience* **25**(1), 471–490 (2002).

[94] Lütolf, S., Radtke, F., Aguet, M., Suter, U., and Taylor, V. Notch1 is required for neuronal and glial differentiation in the cerebellum. *Development* **129**(2), 373–385, January (2002).

[95] Reynolds, B. A. and Weiss, S. Clonal and Population Analyses Demonstrate That an EGF-Responsive Mammalian Embryonic CNS Precursor Is a Stem Cell. *Developmental Biology* **175**(1), 1–13, April (1996).

[96] Weiss, S., Reynolds, B. A., Vescovi, A. L., Morshead, C., Craig, C. G., et al. Is there a neural stem cell in the mammalian forebrain? *Trends in Neurosciences* **19**(9), 387–393 (1996).

[97] Okano, H. and Temple, S. Cell types to order: temporal specification of CNS stem cells. *Current Opinion in Neurobiology* **19**(2), 112–119, April (2009).

[98] Temple, S. The development of neural stem cells. *Nature* **414**(6859), 112–117, November (2001).

[99] Le Dréau, G. and Martí, E. Dorsal–ventral patterning of the neural tube: A tale of three signals. *Developmental Neurobiology* **72**(12), 1471–1481 (2012).

[100] Fuccillo, M., Joyner, A. L., and Fishell, G. Morphogen to mitogen: the multiple roles of hedgehog signalling in vertebrate neural development. *Nature Reviews Neuroscience* **7**(10), 772–783, October (2006).

[101] Shen, Q., Qian, X., Capela, A., and Temple, S. Stem cells in the embryonic cerebral cortex: their role in histogenesis and patterning. *Journal of Neurobiology* **36**(2), 162–174, August (1998).

[102] Shen, Q., Wang, Y., Dimos, J. T., Fasano, C. A., Phoenix, T. N., et al. The timing of cortical neurogenesis is encoded within lineages of individual progenitor cells. *Nature Neuroscience* **9**(6), 743–751, June (2006).

[103] Jessell, T. M. Neuronal specification in the spinal cord: inductive signals and transcriptional codes. *Nature Reviews. Genetics* **1**(1), 20–29, October (2000).

[104] Shirasaki, R. and Pfaff, S. L. Transcriptional Codes and the Control of Neuronal Identity. *Annual Review of Neuroscience* **25**(1), 251–281 (2002).

[105] Philippidou, P. and Dasen, J. S. Hox Genes: Choreographers in Neural Development, Architects of Circuit Organization. *Neuron* **80**(1), 12–34, February (2013).

[106] Liu, A. and Joyner, A. L. Early Anterior/Posterior Patterning of the Midbrain and Cerebellum. *Annual Review of Neuroscience* **24**(1), 869–896 (2001).

[107] Puelles, L. and Rubenstein, J. L. R. Forebrain gene expression domains and the evolving prosomeric model. *Trends in Neurosciences* **26**(9), 469–476, September (2003).

[108] Cambronero, F. and Puelles, L. Rostrocaudal nuclear relationships in the avian medulla oblongata: A fate map with quail chick chimeras. *The Journal of Comparative Neurology* **427**(4), 522–545, November (2000).

[109] Marín, F., Aroca, P., and Puelles, L. Hox gene colinear expression in the avian medulla oblongata is correlated with pseudorhombomeric domains. *Developmental Biology* **323**(2), 230–247, November (2008).

[110] Holstege, J. C., de Graaff, W., Hossaini, M., Cano, S. C., Jaarsma, D., et al. Loss of Hoxb8 alters spinal dorsal laminae and sensory responses in mice. *Proceedings of the National Academy of Sciences of the United States of America* **105**(17), 6338–6343, April (2008).

[111] Lumsden, A. and Krumlauf, R. Patterning the vertebrate neuraxis. *Science (New York, N.Y.)* **274**(5290), 1109–1115, November (1996).

[112] Sieber, M. A., Storm, R., Martinez-de-la Torre, M., Müller, T., Wende, H., et al. Lbx1 Acts as a Selector Gene in the Fate Determination of Somatosensory and Viscerosensory Relay Neurons in the Hindbrain. *The Journal of Neuroscience* **27**(18), 4902–4909, May (2007).

[113] Wizenmann, A. and Lumsden, A. Segregation of Rhombomeres by Differential Chemoaffinity. *Molecular and Cellular Neuroscience* **9**(5–6), 448–459 (1997).

[114] Joyner, A. L. Engrailed, Wnt and Pax genes regulate midbrain-hindbrain development. *Trends in Genetics* **12**(1), 15–20, January (1996).

[115] Wassef, M. and Joyner, A. L. Early mesencephalon/metencephalon patterning and development of the cerebellum. *Perspectives on Developmental Neurobiology* **5**(1), 3–16 (1997).

[116] Liu, A., Losos, K., and Joyner, A. L. FGF8 can activate Gbx2 and transform regions of the rostral mouse brain into a hindbrain fate. *Development* **126**(21), 4827–4838, November (1999).

[117] Wassarman, K. M., Lewandoski, M., Campbell, K., Joyner, A. L., Rubenstein, J. L., et al. Specification of the anterior hindbrain and establishment of a normal mid/hindbrain organizer is dependent on Gbx2 gene function. *Development* **124**(15), 2923–2934, August (1997).

[118] Joyner, A. L., Liu, A., and Millet, S. Otx2, Gbx2 and Fgf8 interact to position and maintain a mid–hindbrain organizer. *Current Opinion in Cell Biology* **12**(6), 736–741, December (2000).

[119] Martinez-Barbera, J. P., Signore, M., Boyl, P. P., Puelles, E., Acampora, D., et al. Regionalisation of anterior neuroectoderm and its competence in responding to forebrain and midbrain inducing activities depend on mutual antagonism between OTX2 and GBX2. *Development* **128**(23), 4789–4800, December (2001).

[120] Kessel, M. and Gruss, P. Murine developmental control genes. *Science (New York, N.Y.)* **249**(4967), 374–379, July (1990).

[121] Grapin-Botton, A., Bonnin, M. A., and Le, N. M. D. Hox gene induction in the neural tube depends on three parameters: competence, signal supply and paralogue group. *Development* **124**(4), 849–859, February (1997).

[122] Mallo, M., Wellik, D. M., and Deschamps, J. Hox genes and regional patterning of the vertebrate body plan. *Developmental Biology* **344**(1), 7–15, August (2010).

[123] McGinnis, W. and Krumlauf, R. Homeobox genes and axial patterning. *Cell* **68**(2), 283–302, January (1992).

[124] Tümpel, S., Wiedemann, L. M., and Krumlauf, R. Chapter 8 Hox Genes and Segmentation of the Vertebrate Hindbrain. In Current Topics in Developmental Biology, Olivier Pourquié, editor, volume Volume 88 of *Hox Genes*, 103–137. Academic Press (2009).

[125] Barrow, J. R., Stadler, H. S., and Capecchi, M. R. Roles of Hoxa1 and Hoxa2 in patterning the early hindbrain of the mouse. *Development* **127**(5), 933–944, March (2000).

[126] Forlani, S., Lawson, K. A., and Deschamps, J. Acquisition of Hox codes during gastrulation and axial elongation in the mouse embryo. *Development* **130**(16), 3807–3819, August (2003).

[127] Cordes, S. P. Molecular genetics of cranial nerve development in mouse. *Nature Reviews Neuroscience* **2**(9), 611–623, September (2001).

[128] Dasen, J. S., Tice, B. C., Brenner-Morton, S., and Jessell, T. M. A Hox Regulatory Network Establishes Motor Neuron Pool Identity and Target-Muscle Connectivity. *Cell* **123**(3), 477–491, November (2005).

[129] Dasen, J. S. and Jessell, T. M. Chapter Six Hox Networks and the Origins of Motor Neuron Diversity. In Current Topics in Developmental Biology, Olivier Pourquié, editor, volume Volume 88 of *Hox Genes*, 169–200. Academic Press (2009).

[130] Davenne, M., Maconochie, M. K., Neun, R., Pattyn, A., Chambon, P., et al. Hoxa2 and Hoxb2 Control Dorsoventral Patterns of Neuronal Development in the Rostral Hindbrain. *Neuron* **22**(4), 677–691, April (1999).

[131] Kessel, M. and Gruss, P. Homeotic transformations of murine vertebrae and concomitant alteration of Hox codes induced by retinoic acid. *Cell* **67**(1), 89–104, October (1991).

[132] Conlon, R. A. and Rossant, J. Exogenous retinoic acid rapidly induces anterior ectopic expression of murine Hox-2 genes in vivo. *Development* **116**(2), 357–368, October (1992).

[133] Marshall, H., Nonchev, S., Sham, M. H., Muchamore, I., Lumsden, A., et al. Retinoic acid alters hindbrain Hox code and induces transformation of rhombomeres 2/3 into a 4/5 identity. *Nature* **360**(6406), 737–741, December (1992).

[134] Marshall, H., Morrison, A., Studer, M., Pöpperl, H., and Krumlauf, R. Retinoids and Hox genes. *The FASEB Journal* **10**(9), 969–978, July (1996).

[135] Dupe, V., Davenne, M., Brocard, J., Dolle, P., Mark, M., et al. In vivo functional analysis of the Hoxa-1 3' retinoic acid response element (3'RARE). *Development* **124**(2), 399–410, January (1997).

[136] Dupé, V. and Lumsden, A. Hindbrain patterning involves graded responses to retinoic acid signalling. *Development* **128**(12), 2199–2208, June (2001).

[137] Glover, J. C., Renaud, J.-S., and Rijli, F. M. Retinoic acid and hindbrain patterning. *Journal of Neurobiology* **66**(7), 705–725 (2006).

[138] de Roos, K., Sonneveld, E., Compaan, B., ten Berge, D., Durston, A. J., et al. Expression of retinoic acid 4-hydroxylase (CYP26) during mouse and Xenopus laevis embryogenesis. *Mechanisms of Development* **82**(1-2), 205–211, April (1999).

[139] Abu-Abed, S., Dollé, P., Metzger, D., Beckett, B., Chambon, P., et al. The retinoic acid-metabolizing enzyme, CYP26a1, is essential for normal hindbrain patterning, vertebral identity, and development of posterior structures. *Genes & Development* **15**(2), 226–240, January (2001).

[140] MacLean, G., Abu-Abed, S., Dollé, P., Tahayato, A., Chambon, P., et al. Cloning of a novel retinoic-acid metabolizing cytochrome P450, Cyp26b1, and comparative expression analysis with Cyp26a1 during early murine development. *Mechanisms of Development* **107**(1-2), 195–201, September (2001).

[141] Abu-Abed, S., MacLean, G., Fraulob, V., Chambon, P., Petkovich, M., et al. Differential expression of the retinoic acid-metabolizing enzymes CYP26a1 and CYP26b1 during murine organogenesis. *Mechanisms of Development* **110**(1–2), 173–177, January (2002).

[142] Tahayato, A., Dollé, P., and Petkovich, M. Cyp26c1 encodes a novel retinoic acid-metabolizing enzyme expressed in the hindbrain, inner ear, first branchial arch and tooth buds during murine development. *Gene Expression Patterns* **3**(4), 449–454, August (2003).

[143] del Corral, R. D., Olivera-Martinez, I., Goriely, A., Gale, E., Maden, M., et al. Opposing FGF and Retinoid Pathways Control Ventral Neural Pattern, Neuronal Differentiation, and Segmentation during Body Axis Extension. *Neuron* **40**(1), 65–79, September (2003).

[144] Ye, W., Shimamura, K., Rubenstein, J. L. R., Hynes, M. A., and Rosenthal, A. FGF and Shh Signals Control Dopaminergic and Serotonergic Cell Fate in the Anterior Neural Plate. *Cell* **93**(5), 755–766, May (1998).

[145] McMahon, A. P. and Bradley, A. The Wnt-1 (int-1) proto-oncogene is required for development of a large region of the mouse brain. *Cell* **62**(6), 1073–1085, September (1990).

[146] Thomas, K. R. and Capecchi, M. R. Targeted disruption of the murine int-1 proto-oncogene resulting in severe abnormalities in midbrain and cerebellar development. *Nature* **346**(6287), 847–850, August (1990).

[147] Liem Jr., K. F., Tremml, G., Roelink, H., and Jessell, T. M. Dorsal differentiation of neural plate cells induced by BMP-mediated signals from epidermal ectoderm. *Cell* **82**(6), 969–979, September (1995).

[148] Guo, S., Brush, J., Teraoka, H., Goddard, A., Wilson, S. W., et al. Development of Noradrenergic Neurons in the Zebrafish Hindbrain Requires BMP, FGF8, and the Homeodomain Protein Soulless/Phox2a. *Neuron* **24**(3), 555–566, November (1999).

[149] Lam, C. S., Sleptsova-Friedrich, I., Munro, A. D., and Korzh, V. SHH and FGF8 play distinct roles during development of noradrenergic neurons in the locus coeruleus of the zebrafish. *Molecular and Cellular Neuroscience* **22**(4), 501–515, April (2003).

[150] Gaufo, G. O., Wu, S., and Capecchi, M. R. Contribution of Hox genes to the diversity of the hindbrain sensory system. *Development* **131**(6), 1259–1266, March (2004).

[151] Tilleman, H., Hakim, V., Novikov, O., Liser, K., Nashelsky, L., et al. Bmp5/7 in concert with the mid-hindbrain organizer control development of noradrenergic locus coeruleus neurons. *Molecular and Cellular Neuroscience* **45**(1), 1–11, September (2010).

[152] Robertson, S. D., Plummer, N. W., de Marchena, J., and Jensen, P. Developmental origins of central norepinephrine neuron diversity. *Nature neuroscience* **16**(8), 1016–1023, August (2013).

[153] McCaffery, P. and Dräger, U. C. High levels of a retinoic acid-generating dehydrogenase in the meso-telencephalic dopamine system. *Proceedings of the National Academy of Sciences of the United States of America* **91**(16), 7772–7776, August (1994).

[154] Simeone, A., Puelles, E., and Acampora, D. The Otx family. *Current Opinion in Genetics & Development* **12**(4), 409–415, August (2002).

[155] Brodski, C., Weisenhorn, D. M. V., Signore, M., Sillaber, I., Oesterheld, M., et al. Location and Size of Dopaminergic and Serotonergic Cell Populations Are Controlled by the Position of the Midbrain–Hindbrain Organizer. *The Journal of Neuroscience* **23**(10), 4199–4207, May (2003).

[156] Puelles, E., Acampora, D., Lacroix, E., Signore, M., Annino, A., et al. Otx dose-dependent integrated control of antero-posterior and dorso-ventral patterning of midbrain. *Nature Neuroscience* **6**(5), 453–460, May (2003).

[157] Puelles, E., Annino, A., Tuorto, F., Usiello, A., Acampora, D., et al. Otx2 regulates the extent, identity and fate of neuronal progenitor domains in the ventral midbrain. *Development* **131**(9), 2037–2048, May (2004).

[158] Jacobs, F. M. J., Smits, S. M., Noorlander, C. W., Oerthel, L. v., Linden, A. J. A. v. d., et al. Retinoic acid counteracts developmental defects in the substantia nigra caused by Pitx3 deficiency. *Development* **134**(14), 2673–2684, July (2007).

[159] Ono, Y., Nakatani, T., Sakamoto, Y., Mizuhara, E., Minaki, Y., et al. Differences in neurogenic potential in floor plate cells along an anteroposterior location: midbrain dopaminergic neurons originate from mesencephalic floor plate cells. *Development* **134**(17), 3213–3225, September (2007).

[160] Omodei, D., Acampora, D., Mancuso, P., Prakash, N., Giovannantonio, L. G. D., et al. Anterior-posterior graded response to Otx2 controls proliferation and differentiation of dopaminergic progenitors in the ventral mesencephalon. *Development* **135**(20), 3459–3470, October (2008).

[161] Simeone, A., Di Salvio, M., Di Giovannantonio, L. G., Acampora, D., Omodei, D., et al. The role of otx2 in adult mesencephalic-diencephalic dopaminergic neurons. *Molecular neurobiology* **43**(2), 107–113, April (2011).

[162] Simon, H. H., Saueressig, H., Wurst, W., Goulding, M. D., and O'Leary, D. D. M. Fate of Midbrain Dopaminergic Neurons Controlled by the Engrailed Genes. *The Journal of Neuroscience* **21**(9), 3126–3134, May (2001).

[163] Wurst, W., Auerbach, A. B., and Joyner, A. L. Multiple developmental defects in Engrailed-1 mutant mice: an early mid-hindbrain deletion and patterning defects in forelimbs and sternum. *Development* **120**(7), 2065–2075, July (1994).

[164] Danielian, P. S. and McMahon, A. P. Engrailed-1 as a target of the Wnt-1 signalling pathway in vertebrate midbrain development. *Nature* **383**(6598), 332–334, September (1996).

[165] Hanks, M., Wurst, W., Anson-Cartwright, L., Auerbach, A. B., and Joyner, A. L. Rescue of the En-1 mutant phenotype by replacement of En-1 with En-2. *Science (New York, N.Y.)* **269**(5224), 679–682, August (1995).

[166] Shamim, H., Mahmood, R., Logan, C., Doherty, P., Lumsden, A., et al. Sequential roles for Fgf4, En1 and Fgf8 in specification and regionalisation of the midbrain. *Development* **126**(5), 945–959, March (1999).

[167] Araki, I. and Nakamura, H. Engrailed defines the position of dorsal di-mesencephalic boundary by repressing diencephalic fate. *Development* **126**(22), 5127–5135, November (1999).

[168] Millet, S., Campbell, K., Epstein, D. J., Losos, K., Harris, E., et al. A role for Gbx2 in repression of Otx2 and positioning the mid/hindbrain organizer. *Nature* **401**(6749), 161–164, September (1999).

[169] Castelo-Branco, G., Wagner, J., Rodriguez, F. J., Kele, J., Sousa, K., et al. Differential regulation of midbrain dopaminergic neuron development by Wnt-1, Wnt-3a, and Wnt-5a. *Proceedings of the National Academy of Sciences of the United States of America* **100**(22), 12747–12752, October (2003).

[170] Roussa, E. and Krieglstein, K. Induction and specification of midbrain dopaminergic cells: focus on SHH, FGF8, and TGF-β. *Cell and Tissue Research* **318**(1), 23–33, October (2004).

[171] Placzek, M. and Briscoe, J. The floor plate: multiple cells, multiple signals. *Nature Reviews Neuroscience* **6**(3), 230–240, March (2005).

[172] Smits, S. M., Burbach, J. P. H., and Smidt, M. P. Developmental origin and fate of meso-diencephalic dopamine neurons. *Progress in Neurobiology* **78**(1), 1–16, January (2006).

[173] Prakash, N., Brodski, C., Naserke, T., Puelles, E., Gogoi, R., et al. A Wnt1-regulated genetic network controls the identity and fate of midbrain-dopaminergic progenitors in vivo. *Development* **133**(1), 89–98, January (2006).

[174] Smidt, M. P. and Burbach, J. P. H. How to make a mesodiencephalic dopaminergic neuron. *Nature Reviews Neuroscience* **8**(1), 21–32, January (2007).

[175] Giovanni, G. D., Matteo, V. D., and Esposito, E. *Birth, Life and Death of Dopaminergic Neurons in the Substantia Nigra.* Springer, , September (2009).

[176] Alves dos Santos, M. T. and Smidt, M. P. En1 and Wnt signaling in midbrain dopaminergic neuronal development. *Neural Development* **6**, 23, May (2011).

[177] Alvarez-Fischer, D., Fuchs, J., Castagner, F., Stettler, O., Massiani-Beaudoin, O., et al. Engrailed protects mouse midbrain dopaminergic neurons against mitochondrial complex I insults. *Nature neuroscience* **14**(10), 1260–1266, October (2011).

[178] Simeone, A., Puelles, E., Omodei, D., Acampora, D., Di Giovannantonio, L. G., et al. Otx genes in neurogenesis of mesencephalic dopaminergic neurons. *Developmental Neurobiology* **71**(8), 665–679, August (2011).

[179] Simeone, A., Acampora, D., Gulisano, M., Stornaiuolo, A., and Boncinelli, E. Nested expression domains of four homeobox genes in developing rostral brain. *Nature* **358**(6388), 687–690, August (1992).

[180] Donkelaar, H. J. t., Cruysberg, J. R. M., Pennings, R., and Lammens, M. Development and Developmental Disorders of the Brain Stem. In *Clinical Neuroembryology*, 321–370. Springer Berlin Heidelberg, January (2014).

[181] Ribes, V. and Briscoe, J. Establishing and Interpreting Graded Sonic Hedgehog Signaling during Vertebrate Neural Tube Patterning: The Role of Negative Feedback. *Cold Spring Harbor Perspectives in Biology* **1**(2), August (2009).

[182] Briscoe, J. and Ericson, J. The specification of neuronal identity by graded sonic hedgehog signalling. *Seminars in Cell & Developmental Biology* **10**(3), 353–362, June (1999).

[183] Briscoe, J. and Ericson, J. Specification of neuronal fates in the ventral neural tube. *Current Opinion in Neurobiology* **11**(1), 43–49, February (2001).

[184] Pattyn, A., Vallstedt, A., Dias, J. M., Sander, M., and Ericson, J. Complementary roles for Nkx6 and Nkx2 class proteins in the establishment of motoneuron identity in the hindbrain. *Development* **130**(17), 4149–4159, September (2003).

[185] Ashe, H. L. and Briscoe, J. The interpretation of morphogen gradients. *Development* **133**(3), 385–394, February (2006).

[186] Ingham, P. W., Nakano, Y., and Seger, C. Mechanisms and functions of Hedgehog signalling across the metazoa. *Nature Reviews Genetics* **12**(6), 393–406, June (2011).

[187] Hynes, M., Stone, D. M., Dowd, M., Pitts-Meek, S., Goddard, A., et al. Control of Cell Pattern in the Neural Tube by the Zinc Finger Transcription Factor and Oncogene Gli-1. *Neuron* **19**(1), 15–26, July (1997).

[188] Briscoe, J., Sussel, L., Serup, P., Hartigan-O'Connor, D., Jessell, T., et al. Homeobox gene Nkx2.2 and specification of neuronal identity by graded Sonic hedgehog signalling. *Nature* **398**(6728), 622–627 (1999).

[189] Logan, C. Y. and Nusse, R. The Wnt Signaling Pathway in Development and Disease. *Annual Review of Cell and Developmental Biology* **20**(1), 781–810 (2004).

[190] Fasano, C. A. and Studer, L. Too much Sonic, too few neurons. *Nature Neuroscience* **12**(2), 107–108, February (2009).

[191] Park, H. L., Bai, C., Platt, K. A., Matise, M. P., Beeghly, A., et al. Mouse Gli1 mutants are viable but have defects in SHH signaling in combination with a Gli2 mutation. *Development (Cambridge, England)* **127**(8), 1593–1605, April (2000).

[192] Matise, M. P., Epstein, D. J., Park, H. L., Platt, K. A., and Joyner, A. L. Gli2 is required for induction of floor plate and adjacent cells, but not most ventral neurons in the mouse central nervous system. *Development* **125**(15), 2759–2770, August (1998).

[193] Blaess, S., Corrales, J. D., and Joyner, A. L. Sonic hedgehog regulates Gli activator and repressor functions with spatial and temporal precision in the mid/hindbrain region. *Development* **133**(9), 1799–1809, May (2006).

[194] Blaess, S., Bodea, G. O., Kabanova, A., Chanet, S., Mugniery, E., et al. Temporal-spatial changes in Sonic Hedgehog expression and signaling reveal different potentials of ventral mesencephalic progenitors to populate distinct ventral midbrain nuclei. *Neural Development* **6**(1), 29, June (2011).

[195] Metzakopian, E., Lin, W., Salmon-Divon, M., Dvinge, H., Andersson, E., et al. Genome-wide characterization of Foxa2 targets reveals upregulation of floor plate genes and repression of ventrolateral genes in midbrain dopaminergic progenitors. *Development* **139**(14), 2625–2634, July (2012).

[196] Zervas, M., Millet, S., Ahn, S., and Joyner, A. L. Cell Behaviors and Genetic Lineages of the Mesencephalon and Rhombomere 1. *Neuron* **43**(3), 345–357, August (2004).

[197] Sasaki, H. and Hogan, B. L. M. HNF-3β as a regulator of floor plate development. *Cell* **76**(1), 103–115, January (1994).

[198] Stamataki, D., Ulloa, F., Tsoni, S. V., Mynett, A., and Briscoe, J. A gradient of Gli activity mediates graded Sonic Hedgehog signaling in the neural tube. *Genes & Development* **19**(5), 626–641, March (2005).

[199] Litingtung, Y. and Chiang, C. Control of Shh activity and signaling in the neural tube. *Developmental Dynamics* **219**(2), 143–154, October (2000).

[200] Briscoe, J., Pierani, A., Jessell, T. M., and Ericson, J. A Homeodomain Protein Code Specifies Progenitor Cell Identity and Neuronal Fate in the Ventral Neural Tube. *Cell* **101**(4), 435–445, May (2000).

[201] Lin, W., Metzakopian, E., Mavromatakis, Y. E., Gao, N., Balaskas, N., et al. Foxa1 and Foxa2 function both upstream of and cooperatively with Lmx1a and Lmx1b in a feedforward loop promoting mesodiencephalic dopaminergic neuron development. *Developmental Biology* **333**(2), 386–396, September (2009).

[202] Ferri, A. L. M., Lin, W., Mavromatakis, Y. E., Wang, J. C., Sasaki, H., et al. Foxa1 and Foxa2 regulate multiple phases of midbrain dopaminergic neuron development in a dosage-dependent manner. *Development* **134**(15), 2761–2769, August (2007).

[203] Kittappa, R., Chang, W. W., Awatramani, R. B., and McKay, R. D. G. The foxa2 Gene Controls the Birth and Spontaneous Degeneration of Dopamine Neurons in Old Age. *PLoS Biol* **5**(12), e325, December (2007).

[204] Bayly, R. D., Brown, C. Y., and Agarwala, S. A novel role for FOXA2 and SHH in organizing midbrain signaling centers. *Developmental Biology* **369**(1), 32–42, September (2012).

[205] Jacob, J., Ferri, A. L., Milton, C., Prin, F., Pla, P., et al. Transcriptional repression coordinates the temporal switch from motor to serotonergic neurogenesis. *Nature Neuroscience* **10**(11), 1433–1439, November (2007).

[206] Smidt, M. P. and van Hooft, J. A. Subset specification of central serotonergic neurons. *Frontiers in Cellular Neuroscience* **7**, October (2013).

[207] Hirsch, M. R., Tiveron, M. C., Guillemot, F., Brunet, J. F., and Goridis, C. Control of noradrenergic differentiation and Phox2a expression by MASH1 in the central and peripheral nervous system. *Development* **125**(4), 599–608, February (1998).

[208] Pattyn, A., Simplicio, N., van Doorninck, J. H., Goridis, C., Guillemot, F., et al. Ascl1/Mash1 is required for the development of central serotonergic neurons. *Nature Neuroscience* **7**(6), 589–595, June (2004).

[209] Pattyn, A., Guillemot, F., and Brunet, J.-F. Delays in neuronal differentiation in Mash1/Ascl1 mutants. *Developmental Biology* **295**(1), 67–75, July (2006).

[210] Hoekstra, E. J., von Oerthel, L., van der Heide, L. P., Kouwenhoven, W. M., Veenvliet, J. V., et al. Lmx1a Encodes a Rostral Set of Mesodiencephalic Dopaminergic Neurons Marked by the Wnt/B-Catenin Signaling Activator R-spondin 2. *PLoS ONE* **8**(9), e74049, September (2013).

[211] Andersson, E., Tryggvason, U., Deng, Q., Friling, S., Alekseenko, Z., et al. Identification of intrinsic determinants of midbrain dopamine neurons. *Cell* **124**(2), 393–405, January (2006).

[212] Deng, Q., Andersson, E., Hedlund, E., Alekseenko, Z., Coppola, E., et al. Specific and integrated roles of Lmx1a, Lmx1b and Phox2a in ventral midbrain. *Development* **138**(16), 3399–3408, August (2011).

[213] Nakatani, T., Kumai, M., Mizuhara, E., Minaki, Y., and Ono, Y. Lmx1a and Lmx1b cooperate with Foxa2 to coordinate the specification of dopaminergic neurons and control of floor plate cell differentiation in the developing mesencephalon. *Developmental Biology* **339**(1), 101–113, March (2010).

[214] Dai, J.-X., Hu, Z.-L., Shi, M., Guo, C., and Ding, Y.-Q. Postnatal ontogeny of the transcription factor Lmx1b in the mouse central nervous system. *The Journal of Comparative Neurology* **509**(4), 341–355, August (2008).

[215] Matsunaga, E., Katahira, T., and Nakamura, H. Role of Lmx1b and Wnt1 in mesencephalon and metencephalon. *Development* **129**(22), 5269–5277, November (2002).

[216] Smidt, M. P., Asbreuk, C. H. J., Cox, J. J., Chen, H., Johnson, R. L., et al. A second independent pathway for development of mesencephalic dopaminergic neurons requires Lmx1b. *Nature Neuroscience* **3**(4), 337–341, April (2000).

[217] Cheng, L., Chen, C.-L., Luo, P., Tan, M., Qiu, M., et al. Lmx1b, Pet-1, and Nkx2.2 Coordinately Specify Serotonergic Neurotransmitter Phenotype. *The Journal of Neuroscience* **23**(31), 9961–9967, November (2003).

[218] Ding, Y.-Q., Marklund, U., Yuan, W., Yin, J., Wegman, L., et al. Lmx1b is essential for the development of serotonergic neurons. *Nature Neuroscience* **6**(9), 933–938, September (2003).

[219] Tsarovina, K., Pattyn, A., Stubbusch, J., Müller, F., Wees, J. v. d., et al. Essential role of Gata transcription factors in sympathetic neuron. *Development* **131**(19), 4775–4786, October (2004).

[220] Craven, S. E., Lim, K.-C., Ye, W., Engel, J. D., Sauvage, F. d., et al. Gata2 specifies serotonergic neurons downstream of sonic hedgehog. *Development* **131**(5), 1165–1173, March (2004).

[221] van Doorninck, J. H., van Der Wees, J., Karis, A., Goedknegt, E., Engel, J. D., et al. GATA-3 is involved in the development of serotonergic neurons in the caudal raphe nuclei. *The Journal of Neuroscience: The Official Journal of the Society for Neuroscience* **19**(12), RC12, June (1999).

[222] Morin, X., Cremer, H., Hirsch, M.-R., Kapur, R. P., Goridis, C., et al. Defects in Sensory and Autonomic Ganglia and Absence of Locus Coeruleus in Mice Deficient for the Homeobox Gene Phox2a. *Neuron* **18**(3), 411–423, March (1997).

[223] Brunet, J.-F. and Pattyn, A. Phox2 genes — from patterning to connectivity. *Current Opinion in Genetics & Development* **12**(4), 435–440, August (2002).

[224] Pattyn, A., Hirsch, M., Goridis, C., and Brunet, J. F. Control of hindbrain motor neuron differentiation by the homeobox gene Phox2b. *Development* **127**(7), 1349–1358, April (2000).

[225] Pattyn, A., Morin, X., Cremer, H., Goridis, C., and Brunet, J. F. Expression and interactions of the two closely related homeobox genes Phox2a and Phox2b during neurogenesis. *Development* **124**(20), 4065–4075, October (1997).

[226] Tiveron, M.-C., Hirsch, M.-R., and Brunet, J.-F. The Expression Pattern of the Transcription Factor Phox2 Delineates Synaptic Pathways of the Autonomic Nervous System. *The Journal of Neuroscience* **16**(23), 7649–7660, December (1996).

[227] Qian, Y., Fritzsch, B., Shirasawa, S., Chen, C.-L., Choi, Y., et al. Formation of brainstem (nor)adrenergic centers and first-order relay visceral sensory neurons is dependent on homeodomain protein Rnx/Tlx3. *Genes & Development* **15**(19), 2533–2545, October (2001).

[228] Abeliovich, A. and Hammond, R. Midbrain dopamine neuron differentiation: Factors and fates. *Developmental Biology* **304**(2), 447–454, April (2007).

[229] Pabst, O., Rummelies, J., Winter, B., and Arnold, H.-H. Targeted disruption of the homeobox gene Nkx2.9 reveals a role in development of the spinal accessory nerve. *Development* **130**(6), 1193–1202, March (2003).

[230] Ericson, J., Morton, S., Kawakami, A., Roelink, H., and Jessell, T. M. Two Critical Periods of Sonic Hedgehog Signaling Required for the Specification of Motor Neuron Identity. *Cell* **87**(4), 661–673, November (1996).

[231] Vallstedt, A., Muhr, J., Pattyn, A., Pierani, A., Mendelsohn, M., et al. Different Levels of Repressor Activity Assign Redundant and Specific Roles to Nkx6 Genes in Motor Neuron and Interneuron Specification. *Neuron* **31**(5), 743–755, September (2001).

[232] Sander, M., Paydar, S., Ericson, J., Briscoe, J., Berber, E., et al. Ventral neural patterning by Nkx homeobox genes: Nkx6.1 controls somatic motor neuron and ventral interneuron fates. *Genes & Development* **14**(17), 2134–2139, September (2000).

[233] Müller, M., Jabs, N., Lork, D. E., Fritzsch, B., and Sander, M. Nkx6.1 controls migration and axon pathfinding of cranial branchio-motoneurons. *Development* **130**(23), 5815–5826, December (2003).

[234] Kele, J., Simplicio, N., Ferri, A. L. M., Mira, H., Guillemot, F., et al. Neurogenin 2 is required for the development of ventral midbrain dopaminergic neurons. *Development* **133**(3), 495–505, February (2006).

[235] Andersson, E., Jensen, J. B., Parmar, M., Guillemot, F., and Björklund, A. Development of the mesencephalic dopaminergic neuron system is compromised in the absence of neurogenin 2. *Development* **133**(3), 507–516, February (2006).

[236] Lahti, L., Saarimäki-Vire, J., Rita, H., and Partanen, J. FGF signaling gradient maintains symmetrical proliferative divisions of midbrain neuronal progenitors. *Developmental Biology* **349**(2), 270–282, January (2011).

[237] Chakrabarty, K., Oerthel, L. V., Hellemons, A., Clotman, F., Espana, A., et al. Genome wide expression profiling of the mesodiencephalic region identifies novel factors involved in early and late dopaminergic development. *Biology Open* **1**(8), 693–704, August (2012).

[238] Sharma, K., Sheng, H. Z., Lettieri, K., Li, H., Karavanov, A., et al. LIM Homeodomain Factors Lhx3 and Lhx4 Assign Subtype Identities for Motor Neurons. *Cell* **95**(6), 817–828, December (1998).

[239] Thor, S., Andersson, S. G. E., Tomlinson, A., and Thomas, J. B. A LIM-homeodomain combinatorial code for motor-neuron pathway selection. *Nature* **397**(6714), 76–80, January (1999).

[240] Tsuchida, T., Ensini, M., Morton, S. B., Baldassare, M., Edlund, T., et al. Topographic organization of embryonic motor neurons defined by expression of LIM homeobox genes. *Cell* **79**(6), 957–970, December (1994).

[241] Arber, S., Han, B., Mendelsohn, M., Smith, M., Jessell, T. M., et al. Requirement for the Homeobox Gene Hb9 in the Consolidation of Motor Neuron Identity. *Neuron* **23**(4), 659–674, August (1999).

[242] Jacob, J., Storm, R., Castro, D. S., Milton, C., Pla, P., et al. Insm1 (IA-1) is an essential component of the regulatory network that specifies monoaminergic neuronal phenotypes in the vertebrate hindbrain. *Development* **136**(14), 2477–2485, July (2009).

[243] Warnecke, M., Oster, H., Revelli, J.-P., Alvarez-Bolado, G., and Eichele, G. Abnormal development of the locus coeruleus in Ear2(Nr2f6)-deficient mice impairs the functionality of the forebrain clock and affects nociception. *Genes & Development* **19**(5), 614–625, March (2005).

[244] Hendricks, T., Francis, N., Fyodorov, D., and Deneris, E. S. The ETS Domain Factor Pet-1 Is an Early and Precise Marker of Central Serotonin Neurons and Interacts with a Conserved Element in Serotonergic Genes. *The Journal of Neuroscience* **19**(23), 10348–10356, December (1999).

[245] Zetterström, R. H., Solomin, L., Jansson, L., Hoffer, B. J., Olson, L., et al. Dopamine Neuron Agenesis in Nurr1-Deficient Mice. *Science* **276**(5310), 248–250, April (1997).

[246] Veenvliet, J. V., Santos, M. T. M. A. d., Kouwenhoven, W. M., Oerthel, L. v., Lim, J. L., et al. Specification of dopaminergic subsets involves interplay of En1 and Pitx3. *Development* **140**(16), 3373–3384, August (2013).

[247] Jacobs, F. M. J., van der Linden, A. J. A., Wang, Y., von Oerthel, L., Sul, H. S., et al. Identification of Dlk1, Ptpru and Klhl1 as novel Nurr1 target genes in meso-diencephalic dopamine neurons. *Development (Cambridge, England)* **136**(14), 2363–2373, July (2009).

[248] Jacobs, F. M. J., Veenvliet, J. V., Almirza, W. H., Hoekstra, E. J., Oerthel, L. v., et al. Retinoic acid-dependent and -independent gene-regulatory pathways of Pitx3 in meso-diencephalic dopaminergic neurons. *Development* **138**(23), 5213–5222, December (2011).

[249] Saucedo-Cardenas, O., Quintana-Hau, J. D., Le, W.-D., Smidt, M. P., Cox, J. J., et al. Nurr1 is essential for the induction of the dopaminergic phenotype and the survival of ventral mesencephalic late dopaminergic precursor neurons. *Proceedings of the National Academy of Sciences* **95**(7), 4013–4018, March (1998).

[250] Smits, S. M., Ponnio, T., Conneely, O. M., Burbach, J. P. H., and Smidt, M. P. Involvement of Nurr1 in specifying the neurotransmitter identity of ventral midbrain dopaminergic neurons. *European Journal of Neuroscience* **18**(7), 1731–1738, October (2003).

[251] Wallén, Å., Zetterström, R. H., Solomin, L., Arvidsson, M., Olson, L., et al. Fate of Mesencephalic AHD2-Expressing Dopamine Progenitor Cells in Nurr1 Mutant Mice. *Experimental Cell Research* **253**(2), 737–746, December (1999).

[252] Johnson, M. M., Michelhaugh, S. K., Bouhamdan, M., Schmidt, C. J., and Bannon, M. J. The Transcription Factor NURR1 Exerts Concentration-Dependent Effects on Target Genes Mediating Distinct Biological Processes. *Frontiers in Neuroscience* **5**, December (2011).

[253] Smidt, M. P., Smits, S. M., Bouwmeester, H., Hamers, F. P. T., Linden, A. J. A. v. d., et al. Early developmental failure of substantia nigra dopamine neurons in mice lacking the homeodomain gene Pitx3. *Development* **131**(5), 1145–1155, March (2004).

[254] Filippi, A., Dürr, K., Ryu, S., Willaredt, M., Holzschuh, J., et al. Expression and function of nr4a2, lmx1b, and pitx3 in zebrafish dopaminergic and noradrenergic neuronal development. *BMC Developmental Biology* **7**, 135, December (2007).

[255] Smidt, M. P., Schaick, H. S. A. v., Lanctôt, C., Tremblay, J. J., Cox, J. J., et al. A homeodomain gene Ptx3 has highly restricted brain expression in mesencephalic dopaminergic neurons. *Proceedings of the National Academy of Sciences* **94**(24), 13305–13310, November (1997).

[256] Munckhof, P. v. d., Luk, K. C., Ste-Marie, L., Montgomery, J., Blanchet, P. J., et al. Pitx3 is required for motor activity and for survival of a subset of midbrain dopaminergic neurons. *Development* **130**(11), 2535–2542, June (2003).

[257] Nunes, I., Tovmasian, L. T., Silva, R. M., Burke, R. E., and Goff, S. P. Pitx3 is required for development of substantia nigra dopaminergic neurons. *Proceedings of the National Academy of Sciences* **100**(7), 4245–4250, April (2003).

[258] Chung, S., Hedlund, E., Hwang, M., Kim, D. W., Shin, B. S., et al. The homeodomain transcription factor Pitx3 facilitates differentiation of mouse embryonic stem cells into AHD2-expressing dopaminergic neurons. *Molecular and Cellular Neuroscience* **28**(2), 241–252, February (2005).

[259] Kraus, F., Haenig, B., and Kispert, A. Cloning and expression analysis of the mouse T-box gene Tbx20. *Mechanisms of Development* **100**(1), 87–91, January (2001).

[260] Novitch, B. G., Chen, A. I., and Jessell, T. M. Coordinate Regulation of Motor Neuron Subtype Identity and Pan-Neuronal Properties by the bHLH Repressor Olig2. *Neuron* **31**(5), 773–789, September (2001).

[261] Zhou, Q. and Anderson, D. J. The bHLH Transcription Factors OLIG2 and OLIG1 Couple Neuronal and Glial Subtype Specification. *Cell* **109**(1), 61–73, April (2002).
